# Viral chimeras decrypt the role of enterovirus capsid proteins in viral tropism, acid sensitivity and optimal growth temperature

**DOI:** 10.1371/journal.ppat.1006962

**Published:** 2018-04-09

**Authors:** Léna Royston, Manel Essaidi-Laziosi, Francisco J. Pérez-Rodríguez, Isabelle Piuz, Johan Geiser, Karl-Heinz Krause, Song Huang, Samuel Constant, Laurent Kaiser, Dominique Garcin, Caroline Tapparel

**Affiliations:** 1 University of Geneva Faculty of Medicine, Department of Microbiology and Molecular Medicine, 1 Rue Michel-Servet, Geneva, Switzerland; 2 University of Geneva Faculty of Medicine, Department of Pathology and Immunology, 1 Rue Michel-Servet, Geneva, Switzerland; 3 Epithelix Sàrl, 18 Chemin des Aulx, Geneva, Switzerland; 4 Laboratory of Virology, Division of Infectious Diseases, University of Geneva Hospitals, 4 Rue Gabrielle Perret-Gentil, Geneva 14, Switzerland; University of Pittsburgh, UNITED STATES

## Abstract

Despite their genetic similarities, enteric and respiratory enteroviruses (EVs) have highly heterogeneous biophysical properties and cause a vast diversity of human pathologies. *In vitro* differences include acid sensitivity, optimal growth temperature and tissue tropism, which reflect a preferential *in vivo* replication in the respiratory or gastrointestinal tract and are thus key determinants of EV virulence. To investigate the underlying cause of these differences, we generated chimeras at the capsid-level between EV-D68 (a respiratory EV) and EV-D94 (an enteric EV). Although some chimeras were nonfunctional, EV-D94 with both the capsid and 2A protease or the capsid only of EV-D68 were both viable. Using this latter construct, we performed several functional assays, which indicated that capsid proteins determine acid sensitivity and tropism in cell lines and in respiratory, intestinal and neural tissues. Additionally, capsid genes were shown to also participate in determining the optimal growth temperature, since EV-D94 temperature adaptation relied on single mutations in VP1, while constructs with EV-D68 capsid could not adapt to higher temperatures. Finally, we demonstrate that EV-D68 maintains residual binding-capacity after acid-treatment despite a loss of infectivity. In contrast, non-structural rather than capsid proteins modulate the innate immune response in tissues. These unique biophysical insights expose another layer in the phenotypic diversity of one of world’s most prevalent pathogens and could aid target selection for vaccine or antiviral development.

## Author summary

Enteroviruses (EV) are one of the most frequent human pathogens worldwide, causing a broad spectrum of diseases, ranging from the common cold to fatal flaccid paralysis. Surprisingly, this vast phenotypic diversity is not reflected at the genetic level, where despite intensive research, the factors contributing to these variations remain poorly understood. By generating novel chimeric viruses derived from a respiratory (EV-D68) and an enteric (EV-D94) EV, we highlight the crucial role of capsid proteins in acid sensitivity, *in vitro* tropism and optimal growth temperature. We also show that while capsid proteins are central to the biophysical viability of EV, they elicit only minor effects on the innate immune response. This work identifies key players in the pathogenesis of these highly prevalent viruses, which not only improves our understanding of their biology, but may also better guide the selection of new targets for vaccine and antiviral innovations.

## Introduction

Enteroviruses (EVs) represent one of the leading causes of human disease worldwide and are associated with a broad spectrum of clinically distinct syndromes. While most infections are benign or asymptomatic, a small proportion may lead to life-threatening disease. Apart from the successful vaccines against poliovirus, there are currently no efficient antiviral treatments or prophylaxes against these common and potentially fatal pathogens.

**Fig 1:**
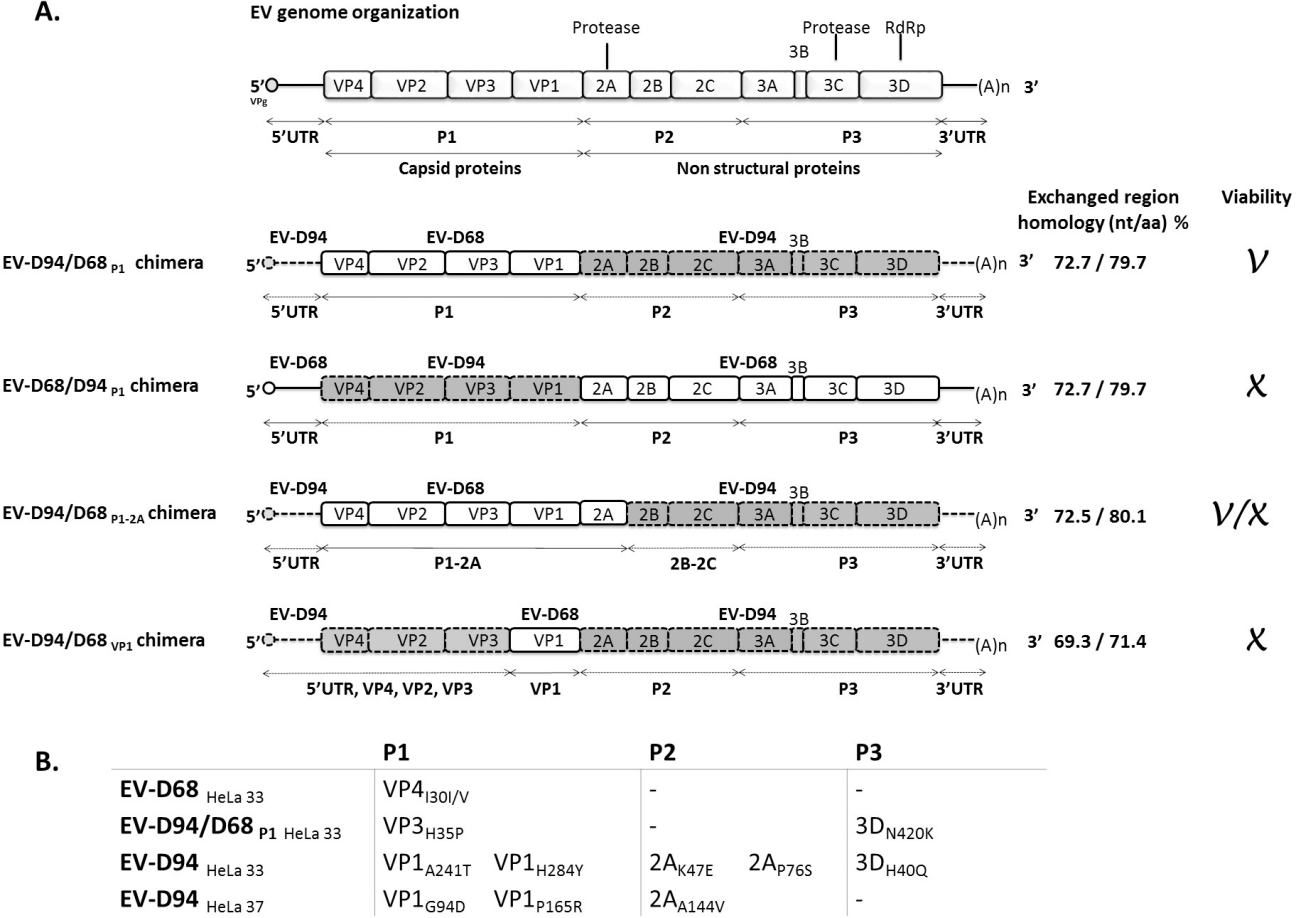
**A.** Schematic representation of the artificially engineered chimeric EV-D68/EV-D94 viruses. Construct names are indicated on the left. EV-D68 regions are represented by white boxes; EV-D94 regions are in grey. Results obtained upon transfection and passage in HeLa cells are indicated on the right, as is the percentage of nucleotide and amino acid sequence identity between the exchanged regions. V, viable construct; X, non-viable construct; V/X, viable unfit construct. **B.** Non-synonymous adaptation mutations observed in viral stocks after 6 passages in HeLa cells. Sequencing was performed from nt 45 to 7344. Production of viral stocks by transfection at both 33°C and 37°C was attempted for all viruses, but only EV-D94 could be recovered at 37°C. For EV-D94, the stock subsequently used for phenotypic assessment is the one prepared at 33°C. HeLa 33 and HeLa 37: viral stocks transfected and amplified at 33°C and 37°C respectively.

Classified in the *Picornaviridae* family, the *Enterovirus* genus is composed of over 300 different genotypes distributed among 13 species (EV-A to EV-J and RV-A to RV-C), seven of which include human pathogens [1]. These divisions are based primarily on nucleotide and amino acid homology rather than the phenotypic presentation of human infection. Their positive-stranded RNA genome of 7’200 to 7’500 nucleotides is packed into a 30 nm icosahedral capsid. The 5’ end of the genome is covalently linked to the small viral protein, VPg, and contains a highly structured untranslated region (5’UTR) composed of *cis*-acting elements necessary for replication and translation. An internal ribosomal entry site (IRES) is required for cap-independent translation, which gives rise to a precursor polyprotein that is subsequently cleaved into 11 mature proteins by viral proteases. This precursor polyprotein encompasses 3 major regions (P1-P3). The P1 region encodes capsid proteins (1A to 1D or VP4 to VP1), while the P2 and P3 regions encode non-structural proteins (2A to 2C and 3A to 3D). The 3’UTR is marked by a poly-A tail and contains elements involved in the regulation of replication [2] (Fig 1).

Although closely related at a genetic level, viruses within the EV genus have distinct transmission routes and replication sites. They also display remarkably different phenotypic characteristics and associated clinical syndromes [3, 4]. Based on their primary replication site, the respiratory or gastrointestinal mucosa, EVs can be divided in respiratory EVs (which also include all rhinovirus (RV) types) and enteric EVs, although gastrointestinal diseases are rarely caused by EV infections [3]. Respiratory EVs are transmitted via the respiratory route, have an infectious tropism restricted to the respiratory tract and rarely disseminate. By contrast, enteric EVs are mostly transmitted via the fecal-oral route, replicate predominantly in the gastrointestinal mucosa from where they can disseminate and infect a wide range of different organs, including the central nervous system. Differences between these 2 groups of viruses also exist *in vitro*: for example, unlike enteric EVs, respiratory EVs exhibit particle instability at low pH, a lower optimal replication temperature, and a restricted cell tropism [5].

Despite intensive research, the genetic roots of this phenotypic variability are still poorly understood. Acid treatment of RVs was shown to result in a loss of infectivity due to a conformational change in the capsid proteins, leading to the expulsion of VP4 [6]. Studies conducted in the 1970’s revealed that acidification followed by neutralization of RV particles produced 2 subviral components (“A”, containing RNA without VP4 and “B”, with neither RNA nor VP4): a transformation that made the RV particles defective in attachment [7]. There are numerous determinants of viral tropism, but as capsid proteins contain the receptor-binding site, they are thought to play a significant role. Other viral proteins potentially important in this process are those interacting with the host innate defenses as well as elements of the translation and replication machinery [8]. With regard to optimal growth temperature, various mutations across the genome have been described in cold-adapted viruses and shown to impact the growth of EVs *in vivo*; however, the exact genomic regions providing resistance to higher body temperatures have not yet been defined [9, 10]. Similar uncertainty exists regarding the determinants of the diversity in immune responses and complex immune evasion strategies between the various EVs. While innate pattern recognition and evasion strategies have been linked to distinct viral proteins such as 2A, 2C and 3C [11], the precise roles of structural and non-structural proteins in these processes are yet to be elucidated.

The aim of this work is to identify the genetic regions responsible for differences in key determinants of viral viability (namely, acid sensitivity, optimal growth temperature, tissue tropism and induction of innate immunity) through the creation of unique viral chimeras. We thus exchanged the viral capsid proteins between respiratory and enteric EVs. As only intraspecies chimeric combinations are viable at the polyprotein level [12], we selected 2 viruses from the same species that are different in acid sensitivity and tissue tropism: EV-D68 as a representative of respiratory EVs [13], and EV-D94 as a representative of enteric EVs [14]. Unlike most non-RVs EVs, EV-D68 virions are acid-labile, replicate optimally at low temperatures, are spread mainly via the respiratory route and cause mild to severe respiratory disease [13, 15]. Because of their physicochemical properties reminiscent of RVs, they were initially misclassified as human RV-87 [13, 16]. Other EVs with a preferred respiratory tropism such as CVA-21 or the recently identified EV-C104, EV-C105, EV-C109, EV-C117 and EV-C118, may also share these properties but as most of these EV-C viruses do not grow in standard culture, their phenotypic characterization remains difficult [3]. While recent EV-D68 isolates have shown neurotropic potential in animal models, the tropism of the original prototypic Fermon strain used in this study is restricted to the respiratory tract [17]. The selected enteric virus, EV-D94 is a recently characterized EV-D type that was isolated concurrently from sewage specimens collected in Egypt and from fecal specimens of acute flaccid paralysis (AFP) patients in the Democratic Republic of the Congo [14]. *In vitro* examinations revealed a stability of the virion under acidic conditions and a remarkably wide tissue and cell tropism [14]. EV-D94 and most EV-D68 strains depend on sialic acid for binding and infection of susceptible cells [18, 19]. ICAM-5/telencephalin was also recently described as a receptor required for entry and replication of sialic acid-dependent and -independent EV-D68 strains [20].

We thus exchanged the capsid regions of EV-D68 and EV-D94 and assessed the viability, tropism (*in vitro* using cell lines and *ex vivo* with reconstituted human tissues), acid sensitivity, temperature adaptation and finally interplay with host innate immunity of the chimeras. Taken together, this work clarifies the diverse biophysical and immunogenic roles of capsid proteins in EV pathogenesis and may guide the search of therapeutic targets.

## Results

### Viability, fitness and adaptation of chimeric EV-D68/EV-D94 viruses

Chimeras were generated by interchanging genomic regions between EV-D68 and EV-D94. Exchanged regions comprised either the capsid region alone (P1), both the capsid and protease regions together (P1 and 2A) or VP1. The following 4 permutations were studied (Fig 1): A) EV-D94/D68_P1_ which integrated the P1 region of EV-D68 into the framework of EV-D94, B) EV-D68/D94_P1_ which integrated the P1 region of EV-D94 into the framework of EV-D68, C) EV-D94/D68_P1-2A_ which integrated both the P1 and 2A regions of EV-D68 into the framework of EV-D94 and finally D) EV-D94/D68_VP1_ with VP1 of EV-D68 integrated into the framework of EV-D94. After *in vitro* transcription and cell transfection, 2 blind passages were performed and virus viability was assessed by cytopathic effect (CPE) examination and confirmed by immunofluorescence (IF). EV-D94/D68_P1_ and EV-D94/D68_P1-2A_ gave rise to viable replicating viruses, causing CPE in HeLa cells; however, although EV-D94/D68_P1_ growth was similar to EV-D94 and EV-D68, only few cells were infected with EV-D94/D68_P1-2A_ and viral loads measured by quantitative PCR after the first passage was more than 100’000 times higher for EV-D94/D68_P1_ than for D94/D68_P1-2A_. The full genomes of EV-D94, EV-D68 and EV-D94/D68_P1_ obtained in HeLa cells after 3 passages were sequenced and several adaptation mutations were observed, particularly for EV-D94 (Fig 1B). Since EV-D94/D68_P1-2A_ was not fully adapted, we did not analyze mutations of the viral stock by sequencing. No other constructs were viable as shown by absence of CPE, negative IF signal and absence of viral RNA amplification measured by RT-qPCR after 2 passages. The non-viability of EV-D68/D94_P1_ was unexpected since the reverse construct was fully fit. To exclude the presence of unnoticed deleterious mutation in the construct, we generated it again using several different approaches; manually, using a fusion PCR-based procedure and also by ordering the full-length construct. A cell adaptation mutation (VP1_H284Y_) observed in the P1 region of the parental EV-D94 virus (Fig 1B) was also inserted in the chimera to try to boost the potentially unfit virus. However, none of these methods produced viable progeny.

### Capsid proteins are the only determinants of acid sensitivity in EVs

**Fig 2:**
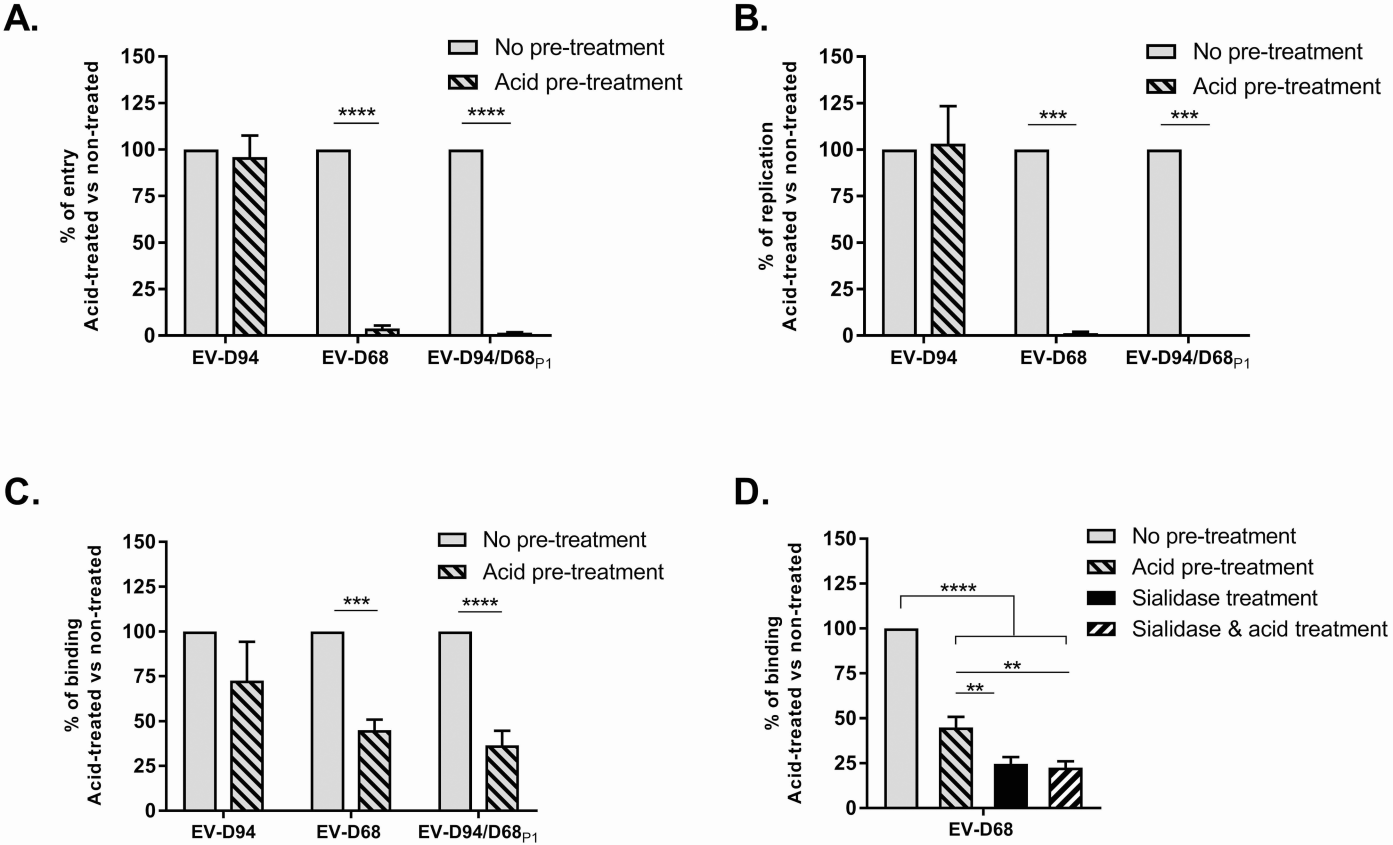
Differential acid sensitivity of EV-D94, EV-D68 and EV-D94/D68_P1_. **A.** Entry assay: viruses (pre-treated or not with acid) were added to cells for 2 hours at 33°C and after extensive washing, viral RNA was extracted for quantification by real-time RT-qPCR. **B.** Replication assay: viruses were added to cells for 1h at 33°C and after extensive washing, cells were further incubated for 24h. Viral RNA was extracted from total cell lysate and quantified. **C.** Binding assay: viruses were added to cells for 1 hour at 4°C to prevent entry. After extensive washing, viral RNA was extracted and quantified. **D.** Binding assay as in C but with cells pretreated with sialidase. For each panel, data are expressed in percentage compared to untreated controls. **P< 0.01. ***P< 0.001. ****P< 0.0001.

Based on viral fitness, EV-D94/D68_P1_ was used in functional assays, and together with its parental viruses (EV-D68 and EV-D94), underwent acid sensitivity testing. A standard protocol was used to test particle stability after acid (pH 3) exposition. While EV-D94 infectivity was unchanged, acid pre-treatment of EV-D68 resulted in a total loss of infectivity. Interestingly, the chimera carrying the EV-D68 P1 region (EV-D94/D68_P1_) was similarly affected by acid pre-treatment. To identify the step of the viral cycle impaired by acid treatment, we performed several assays to test the binding, entry and replication steps based on time post infection (Fig 2). Whereas EV-D94 infectivity was not significantly impaired at any step by acid treatment, entry (Fig 2A) and subsequently replication (Fig 2B) of EV-D68 and EV-D94/D68_P1_ were blocked after acid exposure. In contrast, binding was only partially affected for EV-D68 and EV-D94/D68_P1_ (Fig 2C). To assess the specificity of this residual attachment post acid-treatment, the same binding assay was run on cells pretreated or not with sialidase. Sialidase treatment further inhibited attachment of acid-treated EV-D68 (Fig 2D), indicating a significant and specific binding to sialic acid after acid pretreatment.

### Cellular and tissue tropism of EVs depends exclusively on capsid proteins

#### Differential tropism in cell-lines

Viral replication in several cell lines was tested to assess *in vitro* tropism of EV-D68, EV-D94 and EV-D94/D68_P1_. Numerous human and non-human cell lines originating from various organs were infected with the 3 viruses and infection was assessed by CPE examination and IF (S1 Fig). Infections of HeLa (human cervical carcinoma), RD (human rhabdomyosarcoma), A549 (human lung carcinoma), Caco-2 (human colorectal adenocarcinoma), Vero (African green monkey kidney) and SH-SY5Y (human neuroblastoma) cells were carried out. EV-D94 was able to replicate in all cell types, while EV-D68 replicated only in HeLa, RD, A549 and Caco-2 cells but not in Vero or SH-SY5Y. EV-D94/D68_P1_ exhibited the exact same cellular tropism as EV-D68. The ability of EV-D68 to infect Caco-2 cells, at even higher levels than EV-D94, was not expected considering the colorectal origin of these cells and the restricted respiratory tropism of this virus.

#### Differential tropism in respiratory, intestinal and neural tissues

**Fig 3:**
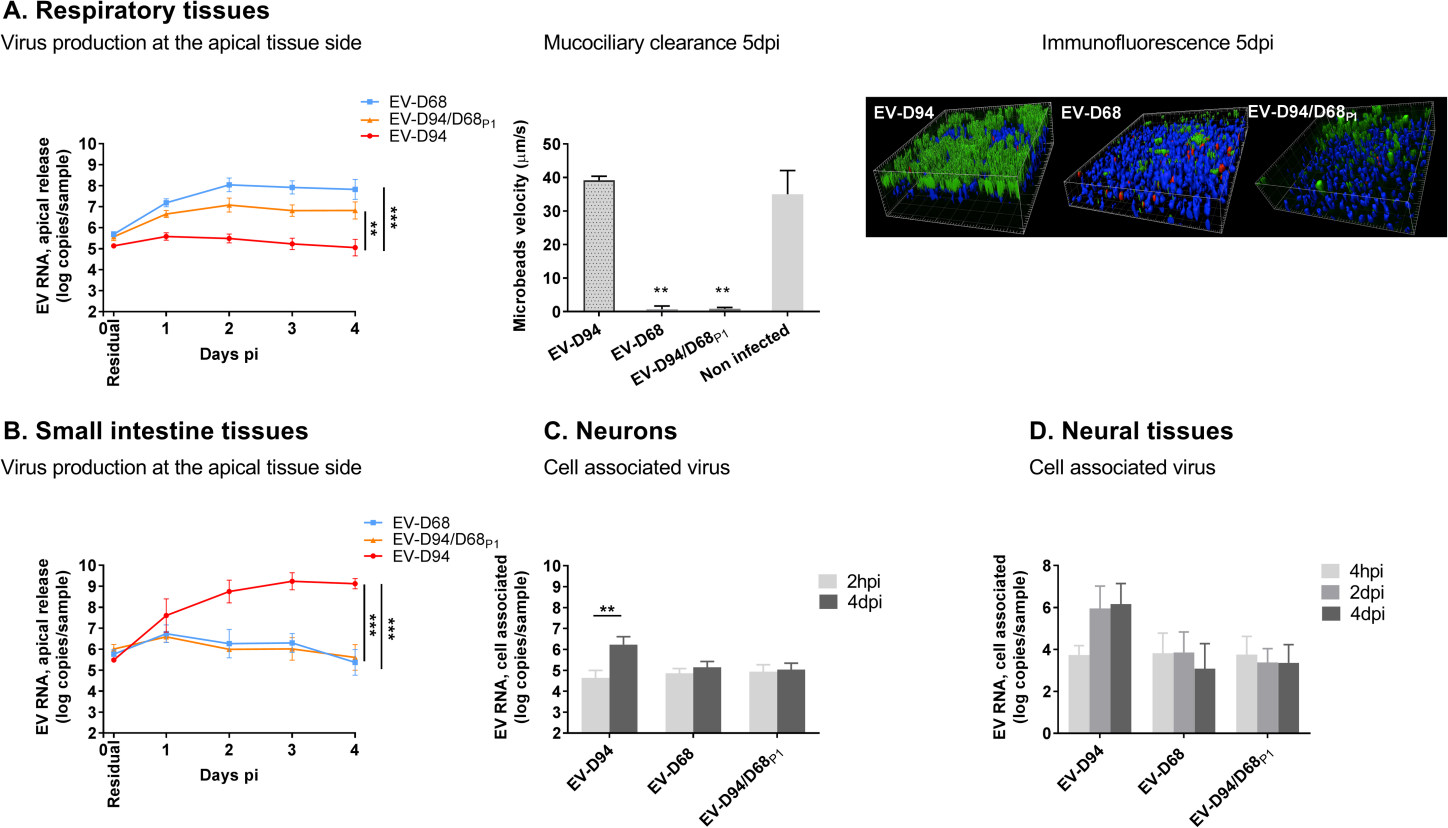
Differential tissue tropism of EV-D94, EV-D68 and EV-D94/D68_P1_. *In vitro* reconstituted human tissues were inoculated with equivalent amount of EV-D94, EV-D68 and EV-D94/D68_P1_ and replication was assessed by RT-qPCR. **A.** 1^st^ Panel: Virus production at the apical tissue side: respiratory tissues were inoculated apically and washed 3 times 4 hpi. Apical samples were collected at the indicated time point for viral RNA quantification. Residual: residual bound virus after 3 washes. 2^nd^ Panel: Mucociliary clearance of infected respiratory tissues assessed by measuring the displacement velocity of polystyrene microbeads applied at the apical side of the tissue 5 dpi. Statistics compare infected and uninfected tissues. 3^rd^ Panel: Immunofluorescence of respiratory tissues 5 dpi with ciliated cells stained in green, viruses in red and cell nuclei in blue. **B.** Small intestine tissues were infected apically and replication was quantified as for respiratory tissues. **C-D.** Neurons (C) and neural tissues (D) were incubated with viral suspension and viral RNA extracted from tissue lysate after inoculation and 2 or 4 days later were compared. **P< 0.01. ***P< 0.001.

To better replicate viral tropism in humans, the same infection assays were performed on complex tissue models *in vitro* reconstituted from human primary cells that mimic the upper respiratory tract, small intestine, neurons or neural tissues composed of neurons and glial cells. For respiratory and gut tissue models grown at an air-liquid interface, cells were infected apically with 10^8^ viral particles (normalised based on RNA quantification) of each viral stock and, after extensive washing at 4 hpi, apical viral release was measured daily as previously described [21, 22]. Residual RNA was measured 4 hpi despite extensive washes in both tissues (Fig 3A and 3B). However, the residual RNA is progressively washed out during the daily collection process, as suggested by the absence of detection of viral RNA at the apical site of respiratory tissues 2dpi with replication deficient viruses (eg UV-irradiated or acid treated viruses) [21].

Between 0.5x10^7^ and 10^8^ viral particles were used to infect primary neurons or engineered neural tissues [23]; but as these cultures are fragile, the viral inoculum was not washed out and replication was assessed by comparing the amount of cell-associated virus directly after inoculation and 4 days later.

In respiratory tissues, viral production peaked between day 1 and 3 for all viruses. However, viral replication of both EV-D68 and EV-D94/D68_P1_ was significantly higher than EV-D94 from day 1 to day 4 (Fig 3A, 1^st^ panel). This higher viral replication was paralleled by an inhibition of mucociliary clearance (MCC) (Fig 3A, 2^nd^ panel). Similarly, we observed a decrease in cilia beating frequency from day 3 in EV-D68 and EV-D94/D68_P1_ infected tissues while neither MCC nor cilia beating were affected by infection with EV-D94. These findings can be linked to the massive loss of ciliated cells observed by IF at 4 dpi caused by EV-D68 and EV-D94/D68_P1_ as opposed to EV-D94 (Fig 3A 3^rd^ panel).

The opposite situation occurred in intestinal tissues where viral loads measured 4 dpi for EV-D94 were around 4 log higher than those of EV-D68 (Fig 3B). Once again, EV-D94/D68_P1_ exhibited the same characteristics of its EV-D68 P1 donor parent strain in the intestinal tissue model. The same replication profile was observed in *in vitro*-reconstituted neural tissues, where no increase in cell-associated virus was measured 4 dpi with EV-D68 and the chimera EV-D94/D68_P1_ in either neuron monolayers (Fig 3C) or complex 3D engineered neural tissues (Fig 3D). Interestingly, EV-D94 could infect both tissues, supporting a potential neurotropism of this newly reported EV. The absolute viral loads of EV-D94 measured in the different tissues 4 dpi were significantly higher in intestinal tissues.

### Non-structural proteins modulate IFN induction levels

**Fig 4:**
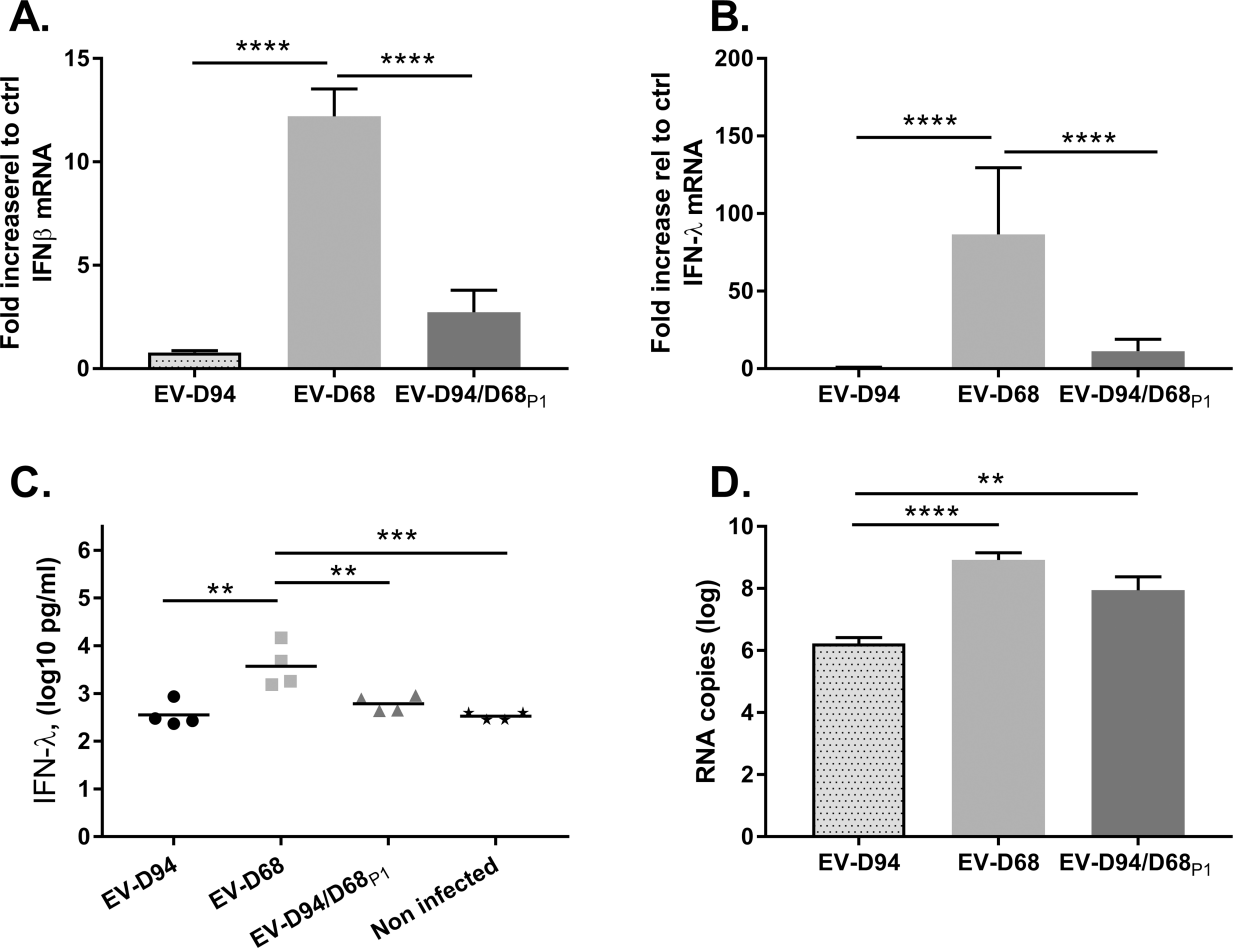
IFN induction in respiratory tissues infected with EV-D94, EV-D68 and EV-D94/D68_P1_. Respiratory tissues were inoculated apically with 10^8^ viral particles (normalised based on RNA quantification) of the indicated virus. Apical washes were performed 4hpi and each day afterwards and tissues were lysed 3 dpi to quantify intracellular RNA (A, B and D). mRNA levels of IFN-β (**A**) and IFN-λ (**B**) in infected versus uninfected tissue were measured by RT-qPCR while IFN-λ protein levels (**C**) were measured in culture medium by ELISA. **D.** Viral loads measured 3 dpi in tissue lysates. **P< 0.01, ***P< 0.001 ****P< 0.0001.

To investigate the implication of structural and non-structural proteins in induction and/or interference with the antiviral response, IFN induction by the different constructs was investigated both in *in vitro-*reconstituted respiratory epithelia and cell lines. IFN induction was not compared in intestinal or neural tissues as both EV-D68 and the chimera replicated poorly in these tissues. First, infected respiratory epithelia were examined 3 dpi at the mRNA level for IFN-β and IFN-λ in tissue lysates by RT-qPCR (Fig 4A and 4B), and at the protein level for IFN-λ in supernatant by ELISA (Fig 4C). IFN-β was not analyzed by ELISA since we previously reported a poor induction of this cytokine in respiratory tissues [22]. Interestingly, IFN induction by EV-D68 and EV-D94 differed significantly both at the protein and mRNA levels; EV-D68 strongly induced both type I and type III IFN, whereas expression levels in EV-D94 and EV-D94/D68_P1_ infected tissues were comparable to uninfected controls. This differential induction did not correlate with differences in viral replication (Fig 4D). Identical infections in RD, A549 and HeLa cells, however, did not induce either type I or type III IFN (S2 Fig).

### Capsid sequences determine sensitivity to high temperatures

To identify the key genetic region implicated in temperature sensitivity, *in vitro* transcripts of EV-D94, EV-D68 and EV-D94/D68_P1_ were transfected at 33°C and 37°C. While the 3 viruses could be recovered after transfection and passages at 33°C, only EV-D94 was recovered after transfection at 37°C. Of note, transfection of EV-D94 and of other unrelated RNAs (such as siRNAs or pathogen associated molecular pattern (PAMP) RNAs for RIG-I) was systematically more efficient at 33°C than at 37°C (D. Garcin personal communication). Titration of viral stocks prepared at 33°C revealed a systematic growth advantage over those prepared at 37°C (S3 Fig).

We then tried to adapt EV-D68 and EV-D94/D68_P1_ stocks obtained at 33°C to higher growth temperature. The two viruses were able to grow at 37°C but with limited fitness and they did not shift their optimal growth temperature to 37°C, despite repeated attempts for EV-D68.

**Fig 5:**
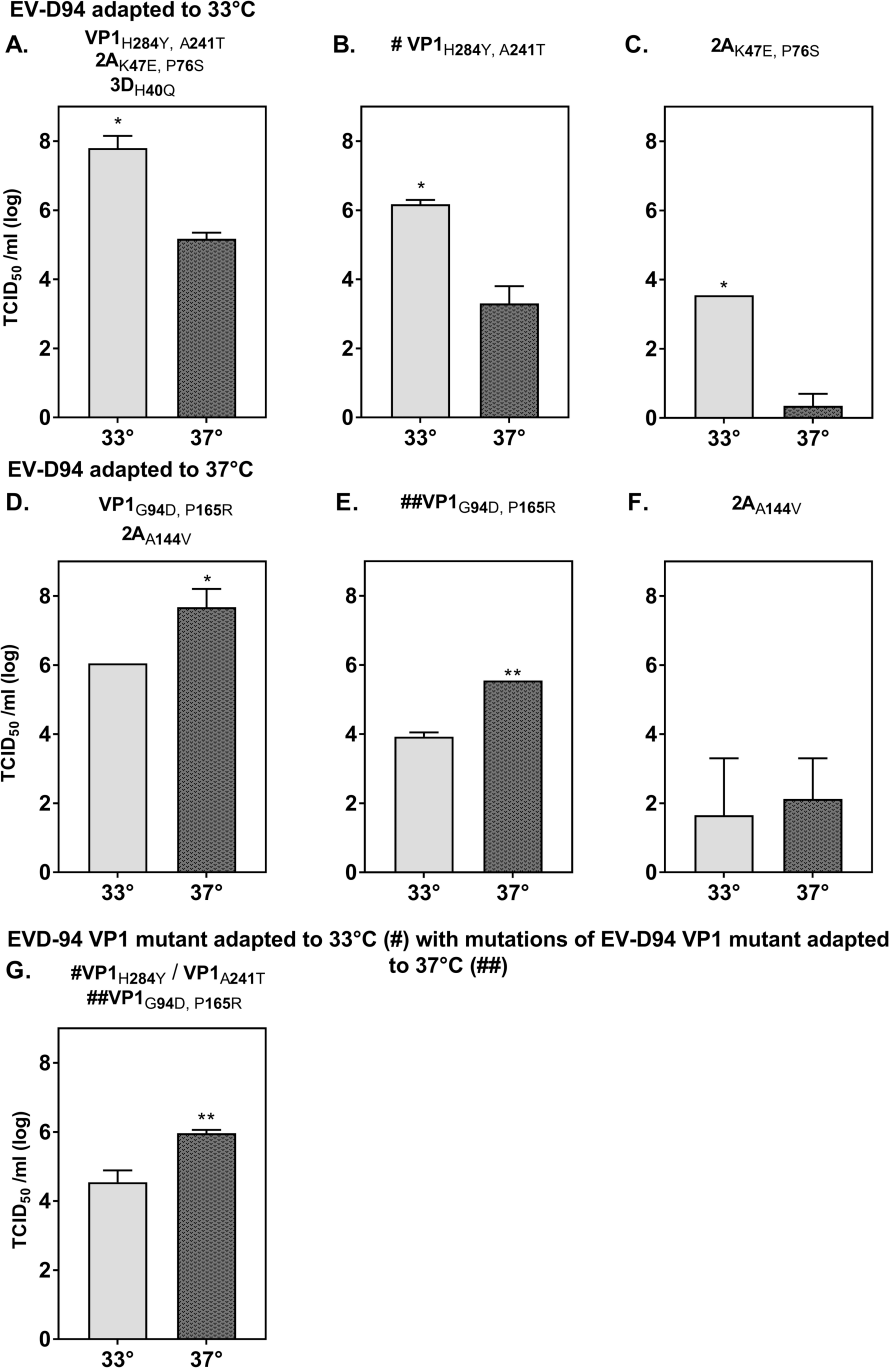
Temperature adaptation of EV-D94. EV-D94 RNA transcribed from the cloned clinical isolate E210 was transfected and passaged 6 times in HeLa cells at either 33°C (“EV-D94 adapted to 33°C”, A) or 37°C (“EV-D94 adapted to 37°C, D). **A.** EV-D94 adapted to 33°C contains 5 non-synonymous mutations relative to the original EV-D94 clone and presents higher titers at 33°C than at 37°C. The VP1 **(B)** or 2A **(C)** mutations were introduced independently in the original infectious clone and the two derivatives retain an optimal growth at 33°C. **D)** EV-D94 adapted to 37°C presents 3 non synonymous mutations relative to the original EV-D94 clone and presents higher titers at 37°C than at 33°C. The VP1 **(E)** or 2A **(F)** mutations were introduced independently in the original infectious clone and only the VP1 mutated virus retains an optimal growth at 37°C. **G.** Mutations in VP1 of EV-D94 adapted to 37°C (E,##) were added to the VP1 mutant adapted to 33°C (B, #) and the viral titers at both temperatures were assessed. *P< 0.05, **P< 0.01.

In contrast, EV-D94 viral stocks adapted to 33°C or 37°C could be obtained in HeLa cells after transfection of the original infectious clone and only 6 passages at these 2 temperatures. These viruses presented different optimal growth temperature not only in HeLa cells (Fig 5) but also in RD, A549 and Vero but to lesser extent (S4 Fig). Whole genome sequencing was performed on these adapted stocks to identify temperature adaptation mutations. Five non-synonymous mutations (VP1_H284Y, A241T_, 2A_K47E, P76S_ and 3D_H40Q_) were observed in EV-D94 adapted to 33°C when compared to the original plasmid construct (Figs 1B and 5A). To find out if some of these mutations were responsible for the improved growth at 33°C, the VP1 (Fig 5B) and 2A (Fig 5C) mutations were introduced independently in the original EV-D94 infectious clone. These 2 derivatives showed a similar optimal growth temperature of 33°C, indicating that none of these mutations was responsible for this phenotype. Since none of these two constructs has the 3D_H40Q_ substitution, an implication of this mutation in the adaptation at 33°C was also excluded. Of note, presence of only the 2A adaptation mutations considerably reduced viral fitness (Fig 5C) implying that the VP1 mutations play a critical role for growth in HeLa cells. To identify mutations resulting in optimal growth at 37°C, the genome of the virus adapted at 37°C was compared with that of the original infectious clone. Two mutations in VP1 (VP1_G94D, P165R_) and 1 mutation in 2A (2A _A144V_) were identified (Fig 5D). Introduction of the 2A mutation alone in the original EV-D94 clone resulted in a drastic fitness loss but a similar growth at 33 and 37°C (Fig 5F). In contrast, introduction of the VP1 mutations reduced viral fitness but did not suppress the growth advantage of the virus at 37°C (Fig 5E), suggesting a key role of these mutations in the adaptation to 37°C. To confirm this hypothesis, these 2 VP1 mutations were introduced in the genetic framework of the virus adapted to 33°C and they drastically shifted its optimal growth temperature from 33°C to 37°C (Fig 5G and S5 Fig).

## Discussion

EV genotypes are co-circulating in humans and are responsible for a wide variety of diseases [4]. The phenotypic diversity of their pathogenesis extends to their biophysical characteristics *in vitro* but is curiously not reflected at the genetic level as is exemplified by members of the EV-D species. By investigating chimeric EV constructs among members of this species, we were able to identify the genomic regions responsible for the key elements of the functional diversity in EV pathogenesis. Using a fully viable chimeric EV-D94 (expressing EV-D68 capsid-encoding genes), we demonstrate that capsid-encoding genes determine viral tropism and acid sensitivity. We also show that EV-D68 capsid presents a major obstacle to adaptation to higher growth temperature while EV-D94 capsid does not. We finally highlight a significant and differential modulation of innate immune response by non-capsid proteins, and thus clarify mechanisms of several layers of EV pathogenesis from biophysical properties to host-pathogen interaction.

The first novel finding in this study is the limited intraspecies compatibility within the EV-D species. Intertypic intraspecies recombination is a frequent event in nature, especially in non-RV EVs. While viable recombinants within the EV-A, -B and -C species have been reported to occur (both *in vitro* as well as in nature [24, 25]), no similar observation has been reported for EV-D. To explore the role of capsid proteins in EV pathogenesis, we created several variants of capsid-chimeras, containing either the full capsid, full capsid and 2A, or VP1 alone. Out of all the constructs, only the exchange of the entire EV-D68 P1 region within the genomic framework of EV-D94 was as viable and fit as the parental viruses. Restrictions of viability were not further explored; however, it may be hypothesised that it was due to the highly ordered structure of icosahedral capsids where mosaic capsids constructions (within P1), can be expected to be poorly viable as was revealed in previous *in vitro* studies on poliovirus [26, 27]. Further, recent natural recombination events within P1 are virtually non-existent [28]. The fact that P1 from EV-D68 in the genomic framework of EV-D94 gave rise to a viable chimera, whereas its reverse counterpart did not (despite various methods of fabrication) was more surprising. This chimera could not be made viable even with the addition of cell-adaptation mutations in the capsid-encoding EV-D94 region. One possible explanation for this limitation could be an incompatibility at the encapsidation step, which is controlled by 2C^ATPase^. This enzyme is believed to interact directly with the VP3 capsid protein to incorporate newly synthesised genomes and a highly specific protein-protein interaction has been shown to be an important constraint of recombinant compatibility [29]. Sequencing of our EV-D94/D68_P1_ viral stocks revealed a non-conservative mutation in VP3, mapping to the interior of the viral capsid (S6A Fig) and possibly reflecting a rescue of a defective encapsidation. However, similar compensatory mutations did not occur for the reverse chimera EV-D68/D94_P1_ despite numerous transfection attempts. This suggests that the incompatibility between structural and non-structural proteins of different genotypes may be unilateral. Of note, we did not observe such incompatibilities when we generated intraspecies chimeric RVs [12], although in this case reciprocal chimeras were not created. Other non-structural proteins, such as VPg, also interact with the capsid at critical points of the viral growth cycle [30] and may cause similar limitations to chimeric constructs [31, 32]. Finally, one cannot exclude that unrecognized *cis*-acting elements, critical for EV-D68 replication may lie in the capsid-coding region.

The chimeric virus EV-D94/D68_P1_ exhibited identical acid sensitivity to that of its P1 donor virus, EV-D68, proving the essential involvement of capsid proteins in this biophysical characteristic. While acid treatment completely inhibited viral entry, we showed that sialic-acid dependent binding was still effective. Our observation that binding of acid-treated EV-D68 virions could be further reduced by sialidase cell-treatment contradicts previous studies in RV-A2, concluding that acidity induces a conformational change in the capsid (forming subviral “A” and “B” particles) that makes it incapable of cell binding [7]. However, recent studies with the same virus established that acid-induced conformational changes were confined to particle “A” transformations, involving the release of VP4 and exposure of N-terminal part of VP1, but that it was not sufficient to provoke RNA release [33, 34]. In these conditions, binding is still possible, albeit diminished. Furthermore, the capsid of EV-D68 may present a different conformation change after acidic treatment than the capsid of RV-A2. Nevertheless, despite a retained ability to bind sialic acid, the conformational change induced by acid treatment definitively prevented viral entry. This may rely on the inability of the virus to bind an entry receptor distinct from sialic acid. Alternatively, the irreversible conformational change induced by acidity may preclude proper viral internalisation and/or uncoating.

As the cellular tropism of EV-D68 was completely transferred to the EV-D94/D68_P1_ chimera, we were able to conclude that tropism is probably determined by virus-receptor interactions of the capsid proteins. Both EV-D68 and EV-D94 are known to bind sialic acid on cells [19]; while some sialic acid-independent strains have been reported for EV-D68, the Fermon strain is sialic acid-dependent. Additionally, ICAM-5/telencephalin (a neuron-specific molecule but also largely present in the respiratory tract), has been recently proposed as the entry receptor [18–20]. Our results suggest the existence of a virus-specific receptor (which could be ICAM-5/telencephalin for EV-D68) responsible for the differential tropism observed between EV-D68 and EV-D94. In addition, acid sensitive viruses may have a facilitated uncoating in compartments with low pH when compared to acid-resistant viruses, and thus rely less on conformational changes induced by capsid-receptor interactions. Accordingly, EV-D68 and EV-D94 probably have different entry mechanisms. Additional experiments in diverse cell lines as well as improved knowledge on EV-D receptor usage will be necessary to better define the different entry pathways of EV-D68 and EV-D94.

As until now no animal model is able to accurately replicate the EV pathogenesis in humans, this makes *in vitro*-reconstituted human tissue models essential to EV research [35]. Indeed, even *in vitro* studies have limitations, as can be seen by our observation that the *in vivo* EV cellular tropism of both EV-D68 and EV-D94 was not reflected in their respective immortalized cell lines. Although *ex vivo* models do not fully reproduce the complexity of human physiology, the first steps of infection can be still accurately studied. As expected, EV-D68 proved to be an efficient respiratory virus, reaching 1000-fold higher titres than EV-D94 in respiratory tissues. The fact that these high replication levels were transferred to the EV-D94/D68_P1_ chimera further underlines the essential role of the P1 capsid region in the EV-D68 phenotype. This similarity between EV-D68 and the EV-D94/D68_P1_ chimera was also seen in ciliated cell toxicity and impaired mucociliary clearance, which is in line with our recently published work on these tissues [22]. Interestingly, EV-D94 did not have the same repercussions and as innate immunity induction of EV-D94/D68_P1_ was comparable to EV-D94, replication levels rather than collateral damage by the host immune response likely explain the pathogenesis observed.

In line with the expected tropism of EV-D94, this virus was able to infect both neural and gastrointestinal tissues, supporting its probable role in AFP cases in humans. While a potential causal role of EV-D68 in AFP has been suggested by epidemiological studies, recent murine models have suggested that only recently emerged strains (and not the Fermon prototypic strain used in this study), have this neuroinvasion ability [17] which was confirmed in our study, where neither EV-D68 nor the EV-D94/D68_P1_ chimera were able to efficiently infect the neural tissue models. Of note, absolute replication levels vary considerably among tissue types. Though these values are not directly comparable due to different experimental settings linked to differences in tissue composition (monolayer, pseudo-stratified, stratified), EV-D94 was shown to reach the highest viral loads in intestinal tissues. This correlates with preferred replication of enteric EVs in the gastrointestinal tract. Taken together, these experiments show that, in contrast to immortalised cell lines, *ex vivo* model systems are reliable surrogates to investigate the early stages of EV pathogenesis and, more importantly, reveal the central involvement of capsid proteins in determining tissue tropism.

Since respiratory tissues sustain important and comparable replication of both EV-D68 and EV-D94/D68_P1_, studies investigating the innate immune response were standardised in this model. As EV-D68 was the only virus to induce both type I and III IFN, whereas EV-D94 and EV-D94/D68_P1_ did not lead to strong IFN induction either at the protein or the mRNA level, we could conclude that this feature is not determined by the P1 genomic region. Chimeras with exchanges of non-structural proteins could help to identify the elements involved in the innate immune response. Of note, innate immunity was not induced at all in cell lines, showing again the limitations of this model.

Similarly to the other observations, the fact that neither EV-D68 nor EV-D94/D68_P1_ could be transfected or adapted to 37°C, strongly implicates the P1 capsid region in EV thermal stability. Concerning EV-D94, our selected strain (E210) was only passaged once in L20B cells and was thus not adapted to grow in HeLa [14]. Acquisition of improved growth in HeLa cells was nevertheless a very quick process and only 6 passages allowed the emergence of 2 temperature-adapted versions of EV-D94 with 5 mutations accumulated upon growth at 33°C and 3 at 37°C. Different combinations of the 33°C adaptation mutations decreased viral fitness but without changing the virus optimal growth temperature. This indicates that the better growth at 33°C is not associated with specific viral mutations but rather represents a default condition linked to higher tolerance of the cells to viral infection at this temperature. This may be related to reduced innate immunity at cold temperature as has been previously demonstrated [36, 37] and could explain why RNA transfection is more efficient at 33°C also for non-viral RNA molecules such as siRNA or RNA PAMPs. In contrast, with the same approach, we identified 2 mutations in VP1 (D94G and P165R) that were critical for optimal growth in cells at 37°C. G94D maps to the VP1 BC loop, while P165R is located in the canyon (S6B Fig). Both the VP1 BC loop and the canyon are known to be involved in virus adaptation [38, 39] and receptor binding [40] and the BC loop was further shown to modulate CVB3 thermolability [41]. In agreement with these published data, these 2 mutations were also important for viral fitness in HeLa cells and it is not clear whether the 2 phenotypes are related. Of note, we did not repeat independent adaptation at 37°C and cannot confirm that these 2 mutations are the only ones conferring improved fitness at higher temperatures. It is also very likely that in the genomic framework of another EV or in another cell type, other mutations may emerge and fill the same function.

To conclude, using recombinants produced *in vitro* between EV-D68, a respiratory EV and EV-D94, an enteric EV, we proved that capsid proteins are both necessary and sufficient to determine the acid sensitivity and restricted tropism of the respiratory EV, as well as its optimal growth temperature. In contrast, we showed that innate immunity induction is rather modulated by non-structural proteins. The same experimental approach could be effective to explore the role of other EV proteins and exchanges between other pairs of EVs could help to generalize our findings to all EVs. Such approaches open the door to an improved understanding of these highly prevalent but poorly understood pathogens.

## Materials and methods

### Reverse genetics

All primers used in this study are listed in the S1 Table. Fig 1 gives a schematic representation of the different constructs **(i) Infectious clones.** Full genomes of EV-D94 (clinical isolate E210, Genbank *DQ916376*) and EV-D68 (strain Fermon, Genbank *AY426531*) introduced in a pcDNA3.1 and pBMH plasmid, respectively, were purchased from Biomatik (Biomatik, Canada). Silent mutations were added in order to introduce restriction sites in between each gene. A T7 RNA polymerase promoter lacking the last G and a PstI restriction site were added before the viral 5’ end and after the polyA sequence respectively for *in vitro* transcription. **(ii) EV-D94/D68**_**P1**_ **chimeric construction.** The EV-D94/D68_P1_ chimeric construction was obtained by exchanging the P1 region of pcDNA3.1-EV-D94 with that of pBMH-EV-D68 using SfoI and BamHI restriction sites. **(iii) EV-D68/D94**_**P1**_ **chimeric construction.** This construct was obtained by 3 different methods: first, the P1 region of pBMH-EV-D68 was exchanged with that of pcDNA3.1-EV-D94 in the pBMH-EV-D68 vector using SfoI and BamHI restriction sites. Second, PCR amplifications were performed of the 5’UTR of EV-D68 ATCC Fermon strain after reverse transcription of viral RNA extracted from a viral stock (PCR1: primers RV156-RV144), P1 of pcDNA3.1-EV-D94 infectious clone (PCR2: RV158-RV159) and P2/P3/3’UTR of EV-D68 (PCR3: RV160-RV140). The resulting PCR products were gel-purified and PCR1+2 were fused together using nested primers (RV139-146). The fusion PCR product was then fused further with PCR3 (RV157-RV161). The forward primer of the final fusion (RV157) contains a floating tail with the T7 RNA polymerase promoter sequence, whereas the reverse (RV161) contains a floating-24T sequence. The final PCR product was used as template for *in vitro* transcription. Finally, an infectious clone composed of the genome of EV-D68 Fermon strain (Genbank *AY426531*) but the P1 region of EV-D94 (clinical isolate E210, Genbank *DQ916376*) in a pMA-7-Ar vector was purchased from Invitrogen GeneArt Gene Synthesis. **(iv) EV-D94/D68**_**VP1**_**chimeric construction.** 5’UTR-VP3 and P2-P3-3’UTR regions of EV-D94 were PCR amplified from the pcDNA3.1-EV-D94 infectious clone (PCR1: primers RV164-RV170 and PCR3: RV168-RV155, respectively), whereas VP1 region of EV-D68 was PCR-amplified from the EV-D68 ATCC Fermon strain (PCR 2: RV171-RV167). The resulting PCR products were gel-purified. PCR1 and 2 were fused together using primers (RV150-RV153) and the fusion product was fused again with PCR3 (RV157-RV169). **(v) EV-D94/D68**_**P1-2A**_**chimeric construction.** P1-2A region from the EV-D68 ATCC Fermon strain was PCR-amplified with modified forward and reverse primers containing SfoI and AvrII restriction sites, respectively (Ent1.87 and Ent1.88). The resulting PCR product was subcloned in a pCR 2.1-TOPO vector (Thermo Fisher Scientific, Waltham, MA, USA) before subcloning in a pcDNA3.1-EV-D94 vector opened with SfoI and AvrII.

### Temperature-adapted viruses

RNA of EV-D94 adapted to 33°C and 37°C (6 passages) was extracted and reverse-transcribed. PCR amplification of either the P1 or 2A region was performed and the resulting PCR products were cloned in a pCR2.1-TOPO vector (Thermo Fischer Scientific, Waltham, USA). Cloning in the pcDNA3.1-EV-D94 vector was undertaken with either SfoI/BamHI or BamHI/AvrII for P1 or 2A inserts, respectively. Site-directed mutagenesis, as described in [42] was used to add VP1_D94G_, VP1_P165R_ on the 33°C_P1_-adapted EV-D94 mutant with primers RV192-193 and RV194-195. All PCR reactions were performed using the taq Phusion (Thermo Fisher Scientific, Waltham, MA, USA) and absence of mutation was checked by sequencing (Fasteris-DNA sequencing service, Switzerland).

### *In vitro* transcription and transfection

RNA was synthesised using the RiboMAX Large Scale RNA Production System-T7 kit (Promega, USA) either on Pst1 linearized plasmids or on PCR products according to manufacturer’s instruction. 2ug of transcribed RNA were transfected in 6-well plates, each well containing 80% confluent HeLa Ohio cells, using the TransMessenger transfection reagent kit (Qiagen, Germany), at either 33°C or 37°C.

### Cell and viral cultures

RD (human rhabdomyosarcoma, ATCC#CCL-136), Vero (kidney monkey, ATCC#CRL-1587), Caco-2 (human colorectal adenocarcinoma, ATCC#HTB 37), and SH-SY5Y (Human neuroblastoma, ATCC #CRL- 2266) cells were cultured as previously described [39]. A549 (human lung carcinoma, ATCC#CCL-185) were cultured as Vero and RD cells while HeLa Ohio cells (kindly provided by F. H. Hayden, University of Virginia, USA) were grown at 37°C in a 5% CO2 environment in Eagle’s minimum essential medium (Lonza, Switzerland) supplemented with 2 mM L-glutamine, 1μg of amphotericin ml^-1^, 100μg of gentamicin ml^-1^, 20μg of vancomycin ml^-1^, and 10% fetal calf serum (FCS). Parental or chimeric viruses were propagated in these cells in McCoy’s 5A medium (Thermofischer Scientific, USA) supplemented with 2% or 5% FBS and incubated at 33°C or at 37°C, respectively, in a 5% CO2 atmosphere.

### Tissues

MucilAir tissues were obtained from Epithelix (Geneva, Switzerland) and cultured at the air-liquid interface as previously described [21, 22]. Intestinal tissues were purchased at MatTek (Ashland, USA) and cultured at an air-liquid interface according to manufacturer’s instruction in the provided culture medium. Primary neurons and 3D neural tissues were both engineered from pluripotent stem cells and cultured as previously described [23].

### Ethics statement

Respiratory (http://www.epithelix.com/products/mucilair) and EpiIntestinal tissues (https://www.mattek.com/products/epiintestinal/tissues) were respectively ordered from Epithelix and MatTek, two biotechnology companies. There, the tissues are developed from anonymized samples and after Ethical approval. Neural tissues were also developed from anonymized samples and following ethical approval. The study was conducted according to the Declaration of Helsinki on biomedical research (Hong Kong amendment, 1989), and the research protocol was approved by our local ethics committee.

### Viral stocks

Viral stocks were produced by transfection of HeLa Ohio cells incubated at 33°C or 37°C. Seven days after transfection, parental and chimeric viruses’ supernatants and cells were collected, subjected to 3 freeze-thaw cycles, purified and re-passaged in 80% confluent HeLa cells. When cytopathic effect (CPE) was observed or at a maximum 7 dpi, supernatants and cells were collected and subjected to 3 freeze-thaw cycles before a 2^nd^ passage. When CPE was observed, supernatants were collected again and subjected to 3 freeze-thaw cycles, purified and aliquoted. Viral stocks were quantified by qPCR and titration, according to the Reed and Muench method [43]. For temperature-adapted EV-D94 stocks, 3 additional passages were achieved the same way. To check for adaptation to viral culture at different temperatures by sequencing, viral RNA was extracted from the stocks, reverse transcribed and PCR amplified with EV-D94 or EV-D68 specific primers (S1 Table).

### Infection *of in vitro*-reconstituted human respiratory epithelia (MucilAir) and small intestine (EpiIntestinal) tissues

MucilAir and EpiIntestinal tissues were infected apically with 100μl of medium containing 10^8^ RNA copies of each virus. The viral inoculum was washed out 3 times at 4 hpi. After these 3 washes and then every day, 200μl of medium was applied apically for 20 minutes at 33°C (or 37°C for small intestine tissues) for sample collection. Basal medium was also collected daily and replaced with 500μl of fresh medium. RNA was extracted and viral replication quantified by RT-qPCR.

### Infection of human primary neurons and 3D engineered neural tissues

Primary neurons and 3D neural tissues were infected with equivalent amount of each virus (0.5x10^7^ to 10^8^ RNA copies) diluted in 100μl or 40μl of culture medium respectively. Due to tissue fragility, the viral inoculum was not washed out. Tissues (without culture medium) were lysed directly after infection (2 hpi for neurons and 4hpi for 3D neural tissues) and 4 dpi to measure cell associated virus. Total RNA was extracted and viral replication was quantified by RT-qPCR.

### RNA extraction, reverse transcription and real-time RT-PCR quantification

RNA was extracted using the NucliSens easyMAG magnetic beads system (BioMérieux, France) according to the manufacturer’s instructions. Retro-transcription was performed with Superscript II (Invitrogen) and either random hexamer primers (Roche) or oligo(dT) primers (Invitrogen), as described [42]. RT-qPCR was performed using the quantitative Entero/Ge/08 assay as previously described [44] or the EV-D68 specific assay [45] in a one-step format using the QuantiTect Probe RT-PCR Kit (Qiagen, Switzerland) according to the manufacturer's instructions, in a StepOne Applied Biosystems thermocycler. 10-fold dilution series (from 2.5*10^8^ to 2.5*10^5^ copies/ml) of the *in vitro* transcribed full-length pBMH-EV-D68 were used as a quantitative reference standard for each run.

### Immunofluorescence (IF)

Infected and control cells were washed twice with phosphate-buffered saline (PBS), fixed and permeabilized for 20 minutes in a methanol-acetone mixture (1:1) at -20°C and then air dried at room temperature during a few minutes. Incubation with the primary mouse J2 monoclonal antibody (Scicons, Hungary), specific for double-stranded RNA longer than 40 bp, and diluted 1:500 in PBS^—^1% bovine serum albumin was realized for 45 minutes at 37°C. Intensive PBS washings were performed prior to second incubation with fluorescein isothiocyanate (FITC)-conjugated anti-mouse IgG antibody (Light Diagnostics, Merck, USA). Then cells were washed 3 times with PBS, stained with 4,6-diamidino-2-phenylindole at room temperature for 5 minutes and washed with PBS a final time. Coverslips were mounted in Fluoroprep mounting medium (BioMérieux, France) and analyzed using standard microscopy.

### IF of Mucilair tissues

At 5 dpi, tissues were washed 3 times with PBS and fixed with 4% paraformaldehyde at room temperature (RT) for 15 minutes. Tissues were then washed 3 times and permeabilized with Perm/wash buffer (BD, USA), then (after 3 more wash steps) the first antibodies were added and incubated for 1 hour at 37°C. Viral staining was performed using the mAbJ2 diluted 1/500 whereas ciliated cells were stained with the rabbit anti-beta IV tubulin Ab (179504 Abcam, UK, diluted 1/250). The Alexa 594-goat anti-mouse Ab (A11032, Life technologies, USA, diluted 1/3000) and the Alexa 488-goat anti-rabbit Ab (A11008 Life technologies, USA, diluted 1/3000) were used as secondary antibodies and incubated for 1 hour at 37°C. After rinsing with PBS, tissues were stained with DAPI, washed with PBS and mounted onto glass slides in Fluoroprep (BioMérieux, France). Images were acquired with a Zeiss LSM 700 Meta confocal microscope with a 63.6/1.4 objective, processed by Imaris and are presented in 3D projections.

### Mucociliary clearance and cilia beating assessment

Polystyrene microbeads (Sigma 84135) displacement velocity at the apical surface was measured with contrast phase microscopy and image ProsPlus software, as already described [22]. Cilia beating movies were recorded with Sony XCD-U60CR microscope and processed with Sony zcl 142 software and ImageJ.

### Acid sensitivity assay

Based on a standard protocol [46], 20 μl of viral stocks were diluted either in 20 μl of 0.1M citrate buffer pH 3.0 or 0.1M of phosphate buffer pH 7.2 and the mixture was incubated for 1 hour at 37°C. After neutralization by adding 40 μl of 0.1M phosphate buffer pH 7.2 and 120 μl of culture medium, samples were inoculated onto HeLa Ohio cells. **Virus binding assay.** HeLa cells were seeded at 5x10^4^ cells/well in 96-well plates. The following day, medium was removed, cells were washed with cold Hanks' Balanced Salt Solution (HBSS) and 200ul of binding buffer (HBSS containing 0.1% sodium azide and 1% BSA) were added on cells, which were then chilled on ice for 10 minutes. Buffer was removed and 100ul of virus mixture were added. Wells were washed 3 times with 200ul of binding buffer directly after inoculation or after 1 hour incubation on ice to assess residual or specific binding respectively, and after 3 washes with PBS, cells were lysed in 200ul of easyMAG lysis buffer to quantify bound RNA by RT-qPCR. Residual RNA was subtracted to bound RNA and percent of bound RNA was calculated relative to non-treated control for each virus.

### Sialidase assay

α2–3,6,8,9 Neuraminidase A (P0722S, New England Biolabs, USA) was diluted 1:100 in a EDB-0.5%BSA buffer, as described [47], added to cells and incubated for 1 hour at 37°C. Cells were then washed once with PBS and used for the binding assay.

### Interferon induction and interferon (IFN) sensitivity assessment

IFN lambda (IFN-λ1/ λ3, IL-29 /IL-28B) was measured in the apical medium of respiratory tissues by enzyme-linked immunosorbent assay ELISA according to manufacturer’s instructions (DY1598B R&D, USA). mRNA of IFN-β and IFN-λ in tissue lysate were quantified using TaqMan Gene Expression Assay (Hs01103582_s1, ThermoFischer, USA) for IFN-β and with primers and probes described previously for IFN-λ [48]. The IFN Ct values were normalized to those of the RNAse P housekeeping gene (3416844, Applied Biosystems/ThermoFischer, USA). Relative quantification was calculated using the 2^-ΔΔCt^ method [49].

### Statistics

Values are expressed as mean (± SEM). Experiments were done at least in biological duplicates and in each experiment, conditions were run in duplicate except for intestinal and 3D neural tissues where biological replicates were done without intra-experimental replicate. Ordinary one-way or two-way ANOVA tests (without matching or pairing) and multiple comparisons were performed with GraphPad Prism 7.02 software. For Fig 3A and 3B, significance was calculated with t-Tests on the area under the curve (AUC) and SE. For Fig 5, unpaired t-Tests were applied.

## Supporting information

S1 Fig Differential cell tropism of EV-D94, EV-D68 and EV-D94/D68P1. Viral stocks were normalized based on TCID_50_ in HeLa cells (dilutions starting from 10^6^ TCID_50_ were performed) and an endpoint dilution assay was performed in various cell lines. Endpoint dilution (expressed in log) was assessed at 5 dpi.
(TIF)

S2 Fig IFN induction in cell lines. RD, Hela and A549 cells were infected with the indicated virus. For HeLa and RD cells, RNA was extracted from cell lysate 24 hpi and IFNβ and λ mRNAs were quantified by quantitative real-time PCR. The IFN Ct values were normalized to those of the RNAse P housekeeping gene (3416844, Applied Biosystems/ThermoFischer, USA) and relative quantification was calculated using the 2^-ΔΔCt^ method. In A549/pr(IFN-β) GFP reporter cells [50], IFN induction was measured by FACS 18 and 24 hpi. Sendai virus was used as a positive control for IFNβ induction in A549 cells. hpi, hours post infection.
(TIF)

S3 Fig Optimal growth temperature of EV-D94, EV-D68 and EV-D94/D68_P1_. Viral stocks obtained after 3 passages in HeLa cells grown at 33°C were titrated at either 33°C or 37°C. Viral titers (expressed in log) were determined using the TCID_50_ method at 5 dpi. All viruses preferentially replicate at 33°C compared to 37°C. Experiments were run as biological duplicates.
(TIF)

S4 Fig EV-D94 was transfected and passaged 6 times at 33°C or 37°C in HeLa to give rise to EV-D94 adapted to 33°C **(A)** and EV-D94 adapted to 37°C **(B)**. Titration of the two stocks in HeLa, RD, Vero and A549 cells was performed at 33°C and 37°C to define their optimal growth temperature in each cell line.
(TIF)

S5 Fig VP1 G94D and P165R confer an optimal growth at 37°C in HeLa and RD cells. Replication of EV-D94 VP1_G94D,P165R_ **(A)** and EV-D94 VP1_H284Y,A241T,G94D, P165R_ **(B)** in HeLa and RD cells.
(TIF)

S6 Fig Mapping of the capsid residues mutated in EV-D94/D68_P1_ (A) or EV-D94 after amplification in cell culture at different temperatures (B). **A.** Residue VP3_H35_ (highlighted in yellow) is imaged in the context of VP3 (in red) and VP4 (in grey). The interior and exterior of the capsid are indicated. **B.** Mapping of residues mutated in EV-D94 based on homology with EV-D68. Amino acid G94D, P165R, A241T and H284Y in EV-D94 VP1 protein correspond to amino acid S82, L153, E227 and R270 in EV-D68. For A and B, mapping were performed on the available 3D structure of the EV-D68 capsid (PBD accession 4WM8 and 5BNP) thanks to Accelrys Discovery Studio Visualizer 3.5 (D.S. Visualizer, Accelrys Software Inc., San Diego, CA, USA 2012).
(TIF)

S1 Table Primers used in this study. (DOCX)

## References

[1] Picornaviridae website 2017. Available from: http://www.picornaviridae.com/enterovirus/enterovirus.htm.

[2] Pilipenko EV, Poperechny KV, Maslova SV, Melchers WJ, Slot HJ, Agol VI. Cis-element, oriR, involved in the initiation of (-) strand poliovirus RNA: a quasi-globular multi-domain RNA structure maintained by tertiary ('kissing') interactions. The EMBO journal. 1996;15(19):5428–36. . 8895586 PMC452285

[3] Royston L, Tapparel C. Rhinoviruses and Respiratory Enteroviruses: Not as Simple as ABC. Viruses. 2016;8(1). doi: 10.3390/v8010016 . 26761027 10.3390/v8010016 PMC4728576

[4] Tapparel C, Siegrist F, Petty TJ, Kaiser L. Picornavirus and enterovirus diversity with associated human diseases. Infection, genetics and evolution: journal of molecular epidemiology and evolutionary genetics in infectious diseases. 2013;14:282–93. doi: 10.1016/j.meegid.2012.10.016 . 23201849 10.1016/j.meegid.2012.10.016

[5] Fields BN, Knipe DM, Howley PM. Fields virology. Chapter 17: Enteroviruses: polioviruses, coxsackieviruses, echoviruses, and newer enteroviruses Philadelphia: Wolters Kluwer Health/Lippincott Williams & Wilkins; 2013.

[6] Giranda VL, Heinz BA, Oliveira MA, Minor I, Kim KH, Kolatkar PR, et al Acid-induced structural changes in human rhinovirus 14: possible role in uncoating. Proceedings of the National Academy of Sciences of the United States of America. 1992;89(21):10213–7. ; PubMed Central PMCID: PMCPMC50308. 1332036 10.1073/pnas.89.21.10213 PMC50308

[7] Noble J, Lonberg-Holm K. Interactions of components of human rhinovirus type 2 with Hela cells. Virology. 1973;51(2):270–8. . 4348300 10.1016/0042-6822(73)90427-3

[8] Whitton JL, Cornell CT, Feuer R. Host and virus determinants of picornavirus pathogenesis and tropism. Nat Rev Microbiol. 2005;3(10):765–76. doi: 10.1038/nrmicro1284 . 16205710 10.1038/nrmicro1284

[9] Sanders BP, de Los Rios Oakes I, van Hoek V, Bockstal V, Kamphuis T, Uil TG, et al Cold-Adapted Viral Attenuation (CAVA): Highly Temperature Sensitive Polioviruses as Novel Vaccine Strains for a Next Generation Inactivated Poliovirus Vaccine. PLoS pathogens. 2016;12(3):e1005483 doi: 10.1371/journal.ppat.1005483 ; PubMed Central PMCID: PMCPMC4816566. 27032093 10.1371/journal.ppat.1005483 PMC4816566

[10] Arita M, Shimizu H, Nagata N, Ami Y, Suzaki Y, Sata T, et al Temperature-sensitive mutants of enterovirus 71 show attenuation in cynomolgus monkeys. The Journal of general virology. 2005;86(Pt 5):1391–401. doi: 10.1099/vir.0.80784-0 . 15831951 10.1099/vir.0.80784-0

[11] Lei X, Xiao X, Wang J. Innate Immunity Evasion by Enteroviruses: Insights into Virus-Host Interaction. Viruses. 2016;8(1). doi: 10.3390/v8010022 ; PubMed Central PMCID: PMCPMC4728582. 26784219 10.3390/v8010022 PMC4728582

[12] Schibler M, Piuz I, Hao W, Tapparel C. Chimeric rhinoviruses obtained via genetic engineering or artificially induced recombination are viable only if the polyprotein coding sequence derives from the same species. Journal of virology. 2015;89(8):4470–80. doi: 10.1128/JVI.03668-14 ; PubMed Central PMCID: PMC4442373. 25653446 10.1128/JVI.03668-14 PMC4442373

[13] Blomqvist S, Savolainen C, Raman L, Roivainen M, Hovi T. Human rhinovirus 87 and enterovirus 68 represent a unique serotype with rhinovirus and enterovirus features. Journal of clinical microbiology. 2002;40(11):4218–23. doi: 10.1128/JCM.40.11.4218-4223.2002 ; PubMed Central PMCID: PMC139630. 12409401 10.1128/JCM.40.11.4218-4223.2002 PMC139630

[14] Smura TP, Junttila N, Blomqvist S, Norder H, Kaijalainen S, Paananen A, et al Enterovirus 94, a proposed new serotype in human enterovirus species D. The Journal of general virology. 2007;88(Pt 3):849–58. doi: 10.1099/vir.0.82510-0 . 17325357 10.1099/vir.0.82510-0

[15] Oberste MS, Maher K, Schnurr D, Flemister MR, Lovchik JC, Peters H, et al Enterovirus 68 is associated with respiratory illness and shares biological features with both the enteroviruses and the rhinoviruses. The Journal of general virology. 2004;85(Pt 9):2577–84. doi: 10.1099/vir.0.79925-0 . 15302951 10.1099/vir.0.79925-0

[16] Savolainen C, Blomqvist S, Mulders MN, Hovi T. Genetic clustering of all 102 human rhinovirus prototype strains: serotype 87 is close to human enterovirus 70. J Gen Virol. 2002;83(Pt 2):333–40. doi: 10.1099/0022-1317-83-2-333 . 11807226 10.1099/0022-1317-83-2-333

[17] Hixon AM, Yu G, Leser JS, Yagi S, Clarke P, Chiu CY, et al A mouse model of paralytic myelitis caused by enterovirus D68. PLoS pathogens. 2017;13(2):e1006199 doi: 10.1371/journal.ppat.1006199 ; PubMed Central PMCID: PMCPMC5322875. 28231269 10.1371/journal.ppat.1006199 PMC5322875

[18] Liu Y, Sheng J, Baggen J, Meng G, Xiao C, Thibaut HJ, et al Sialic acid-dependent cell entry of human enterovirus D68. Nat Commun. 2015;6:8865 doi: 10.1038/ncomms9865 ; PubMed Central PMCID: PMCPMC4660200. 26563423 10.1038/ncomms9865 PMC4660200

[19] Baggen J, Thibaut HJ, Staring J, Jae LT, Liu Y, Guo H, et al Enterovirus D68 receptor requirements unveiled by haploid genetics. Proceedings of the National Academy of Sciences of the United States of America. 2016;113(5):1399–404. doi: 10.1073/pnas.1524498113 ; PubMed Central PMCID: PMCPMC4747778. 26787879 10.1073/pnas.1524498113 PMC4747778

[20] Wei W, Guo H, Chang J, Yu Y, Liu G, Zhang N, et al ICAM-5/Telencephalin Is a Functional Entry Receptor for Enterovirus D68. Cell Host Microbe. 2016;20(5):631–41. doi: 10.1016/j.chom.2016.09.013 . 27923705 10.1016/j.chom.2016.09.013

[21] Tapparel C, Sobo K, Constant S, Huang S, Van Belle S, Kaiser L. Growth and characterization of different human rhinovirus C types in three-dimensional human airway epithelia reconstituted in vitro. Virology. 2013;446(1–2):1–8. doi: 10.1016/j.virol.2013.06.031 . 24074561 10.1016/j.virol.2013.06.031

[22] Essaidi-Laziosi M, Brito F, Benaoudia S, Royston L, Cagno V, Fernandes-Rocha M, et al Propagation of respiratory viruses in human airway epithelia reveals persistent virus-specific signatures. The Journal of allergy and clinical immunology. 2017 doi: 10.1016/j.jaci.2017.07.018 . 28797733 10.1016/j.jaci.2017.07.018 PMC7112338

[23] Cosset E, Martinez Y, Preynat-Seauve O, Lobrinus JA, Tapparel C, Cordey S, et al Human three-dimensional engineered neural tissue reveals cellular and molecular events following cytomegalovirus infection. Biomaterials. 2015;53:296–308. doi: 10.1016/j.biomaterials.2015.02.094 . 25890728 10.1016/j.biomaterials.2015.02.094

[24] Bessaud M, Joffret ML, Blondel B, Delpeyroux F. Exchanges of genomic domains between poliovirus and other cocirculating species C enteroviruses reveal a high degree of plasticity. Scientific reports. 2016;6:38831 doi: 10.1038/srep38831 ; PubMed Central PMCID: PMCPMC5153852. 27958320 10.1038/srep38831 PMC5153852

[25] Harvala H, Kalimo H, Bergelson J, Stanway G, Hyypia T. Tissue tropism of recombinant coxsackieviruses in an adult mouse model. The Journal of general virology. 2005;86(Pt 7):1897–907. doi: 10.1099/vir.0.80603-0 . 15958668 10.1099/vir.0.80603-0

[26] Burke KL, Dunn G, Ferguson M, Minor PD, Almond JW. Antigen chimaeras of poliovirus as potential new vaccines. Nature. 1988;332(6159):81–2. doi: 10.1038/332081a0 . 2450279 10.1038/332081a0

[27] Murdin AD, Lu HH, Murray MG, Wimmer E. Poliovirus antigenic hybrids simultaneously expressing antigenic determinants from all three serotypes. The Journal of general virology. 1992;73 (Pt 3):607–11. doi: 10.1099/0022-1317-73-3-607 . 1372037 10.1099/0022-1317-73-3-607

[28] Santti J, Hyypia T, Kinnunen L, Salminen M. Evidence of recombination among enteroviruses. Journal of virology. 1999;73(10):8741–9. ; PubMed Central PMCID: PMC112895. 10482628 10.1128/jvi.73.10.8741-8749.1999 PMC112895

[29] Liu Y, Wang C, Mueller S, Paul AV, Wimmer E, Jiang P. Direct interaction between two viral proteins, the nonstructural protein 2C and the capsid protein VP3, is required for enterovirus morphogenesis. PLoS pathogens. 2010;6(8):e1001066 doi: 10.1371/journal.ppat.1001066 ; PubMed Central PMCID: PMCPMC2928791. 20865167 10.1371/journal.ppat.1001066 PMC2928791

[30] Kaiser WJ, Chaudhry Y, Sosnovtsev SV, Goodfellow IG. Analysis of protein-protein interactions in the feline calicivirus replication complex. The Journal of general virology. 2006;87(Pt 2):363–8. doi: 10.1099/vir.0.81456-0 . 16432023 10.1099/vir.0.81456-0

[31] Jiang P, Liu Y, Ma HC, Paul AV, Wimmer E. Picornavirus morphogenesis. Microbiol Mol Biol Rev. 2014;78(3):418–37. doi: 10.1128/MMBR.00012-14 ; PubMed Central PMCID: PMCPMC4187686. 25184560 10.1128/MMBR.00012-14 PMC4187686

[32] Cameron CE, Oh HS, Moustafa IM. Expanding knowledge of P3 proteins in the poliovirus lifecycle. Future Microbiol. 2010;5(6):867–81. doi: 10.2217/fmb.10.40 ; PubMed Central PMCID: PMCPMC2904470. 20521933 10.2217/fmb.10.40 PMC2904470

[33] Bilek G, Matscheko NM, Pickl-Herk A, Weiss VU, Subirats X, Kenndler E, et al Liposomal nanocontainers as models for viral infection: monitoring viral genomic RNA transfer through lipid membranes. Journal of virology. 2011;85(16):8368–75. doi: 10.1128/JVI.00329-11 ; PubMed Central PMCID: PMCPMC3147984. 21680510 10.1128/JVI.00329-11 PMC3147984

[34] Pickl-Herk A, Luque D, Vives-Adrian L, Querol-Audi J, Garriga D, Trus BL, et al Uncoating of common cold virus is preceded by RNA switching as determined by X-ray and cryo-EM analyses of the subviral A-particle. Proceedings of the National Academy of Sciences of the United States of America. 2013;110(50):20063–8. doi: 10.1073/pnas.1312128110 ; PubMed Central PMCID: PMCPMC3864292. 24277846 10.1073/pnas.1312128110 PMC3864292

[35] Wang YF, Yu CK. Animal models of enterovirus 71 infection: applications and limitations. J Biomed Sci. 2014;21:31 doi: 10.1186/1423-0127-21-31 ; PubMed Central PMCID: PMCPMC4013435. 24742252 10.1186/1423-0127-21-31 PMC4013435

[36] Foxman EF, Storer JA, Fitzgerald ME, Wasik BR, Hou L, Zhao H, et al Temperature-dependent innate defense against the common cold virus limits viral replication at warm temperature in mouse airway cells. Proceedings of the National Academy of Sciences of the United States of America. 2015;112(3):827–32. doi: 10.1073/pnas.1411030112 ; PubMed Central PMCID: PMC4311828. 25561542 10.1073/pnas.1411030112 PMC4311828

[37] Foxman EF, Storer JA, Vanaja K, Levchenko A, Iwasaki A. Two interferon-independent double-stranded RNA-induced host defense strategies suppress the common cold virus at warm temperature. Proceedings of the National Academy of Sciences of the United States of America. 2016;113(30):8496–501. doi: 10.1073/pnas.1601942113 ; PubMed Central PMCID: PMCPMC4968739. 27402752 10.1073/pnas.1601942113 PMC4968739

[38] Martin A, Wychowski C, Couderc T, Crainic R, Hogle J, Girard M. Engineering a poliovirus type 2 antigenic site on a type 1 capsid results in a chimaeric virus which is neurovirulent for mice. Embo J. 1988;7(9):2839–47. . 2460345 10.1002/j.1460-2075.1988.tb03140.x PMC457076

[39] Cordey S, Petty TJ, Schibler M, Martinez Y, Gerlach D, van Belle S, et al Identification of site-specific adaptations conferring increased neural cell tropism during human enterovirus 71 infection. PLoS pathogens. 2012;8(7):e1002826 Epub 2012/08/23. doi: 10.1371/journal.ppat.1002826 ; PubMed Central PMCID: PMC3406088. 22910880 10.1371/journal.ppat.1002826 PMC3406088

[40] Rossmann MG, He Y, Kuhn RJ. Picornavirus-receptor interactions. Trends Microbiol. 2002;10(7):324–31. . 12110211 10.1016/s0966-842x(02)02383-1

[41] McPhee F, Zell R, Reimann BY, Hofschneider PH, Kandolf R. Characterization of the N-terminal part of the neutralizing antigenic site I of coxsackievirus B4 by mutation analysis of antigen chimeras. Virus Res. 1994;34(2):139–51. . 7531922 10.1016/0168-1702(94)90096-5

[42] Schibler M, Gerlach D, Martinez Y, Belle SV, Turin L, Kaiser L, et al Experimental human rhinovirus and enterovirus interspecies recombination. The Journal of general virology. 2012;93(Pt 1):93–101. doi: 10.1099/vir.0.035808-0 . 21940413 10.1099/vir.0.035808-0

[43] Flint SJ, Racaniello VR, Rall GF, Skalka AM, Enquist LW. Principles of virology 4th edition. ed. Washington, DC: ASM Press; 2015. volumes p.

[44] Tapparel C, Cordey S, Van Belle S, Turin L, Lee WM, Regamey N, et al New molecular detection tools adapted to emerging rhinoviruses and enteroviruses. Journal of clinical microbiology. 2009;47(6):1742–9. doi: 10.1128/JCM.02339-08 ; PubMed Central PMCID: PMCPMC2691104. 19339471 10.1128/JCM.02339-08 PMC2691104

[45] Royston L, Geiser J, Josset L, Schuffenecker I, Tapparel C. A new real-time RT-PCR targeting VP4-VP2 to detect and quantify enterovirus D68 in respiratory samples. Journal of medical virology. 2017 doi: 10.1002/jmv.24778 . 28169437 10.1002/jmv.24778

[46] Couch RB. Rhinoviruses. New York, N.Y1992. p. 709–29 p.

[47] Malakhov MP, Aschenbrenner LM, Smee DF, Wandersee MK, Sidwell RW, Gubareva LV, et al Sialidase fusion protein as a novel broad-spectrum inhibitor of influenza virus infection. Antimicrob Agents Chemother. 2006;50(4):1470–9. doi: 10.1128/AAC.50.4.1470-1479.2006 ; PubMed Central PMCID: PMCPMC1426979. 16569867 10.1128/AAC.50.4.1470-1479.2006 PMC1426979

[48] Dolganiuc A, Kodys K, Marshall C, Saha B, Zhang S, Bala S, et al Type III interferons, IL-28 and IL-29, are increased in chronic HCV infection and induce myeloid dendritic cell-mediated FoxP3+ regulatory T cells. PloS one. 2012;7(10):e44915 doi: 10.1371/journal.pone.0044915 ; PubMed Central PMCID: PMCPMC3468613. 23071503 10.1371/journal.pone.0044915 PMC3468613

[49] Livak KJ, Schmittgen TD. Analysis of relative gene expression data using real-time quantitative PCR and the 2(-Delta Delta C(T)) Method. Methods. 2001;25(4):402–8. doi: 10.1006/meth.2001.1262 . 11846609 10.1006/meth.2001.1262

[50] Anchisi S, Guerra J, Garcin D. RIG-I ATPase activity and discrimination of self-RNA versus non-self-RNA. mBio. 2015;6(2):e02349 doi: 10.1128/mBio.02349-14 ; PubMed Central PMCID: PMCPMC4358010. 25736886 10.1128/mBio.02349-14 PMC4358010

