# Supplementary figures and images for: Viral chimeras decrypt the role of enterovirus capsid proteins in viral tropism, acid sensitivity and optimal growth temperature
Source: PLoS Pathog. 2018 Apr 9;14(4):e1006962. doi: 10.1371/journal.ppat.1006962 (PMC5908207; doi:10.1371/journal.ppat.1006962)

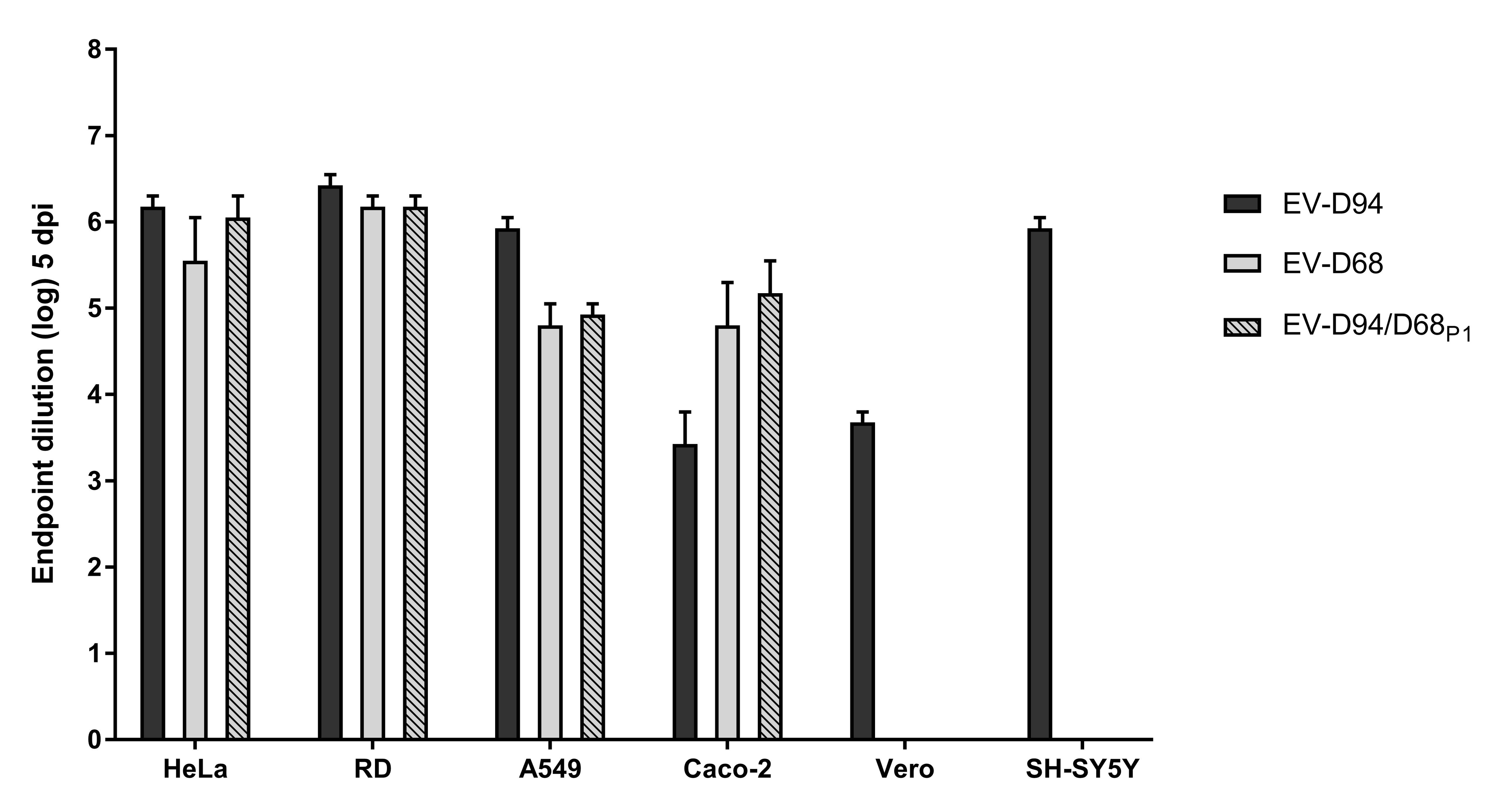

Supplement: S1 Fig — Viral stocks were normalized based on TCID50 in HeLa cells (dilutions starting from 106 TCID50 were performed) and an endpoint dilution assay was performed in various cell lines. Endpoint dilution (expressed in log) was assessed at 5 dpi. (TIF) [file ppat.1006962.s001.tif]

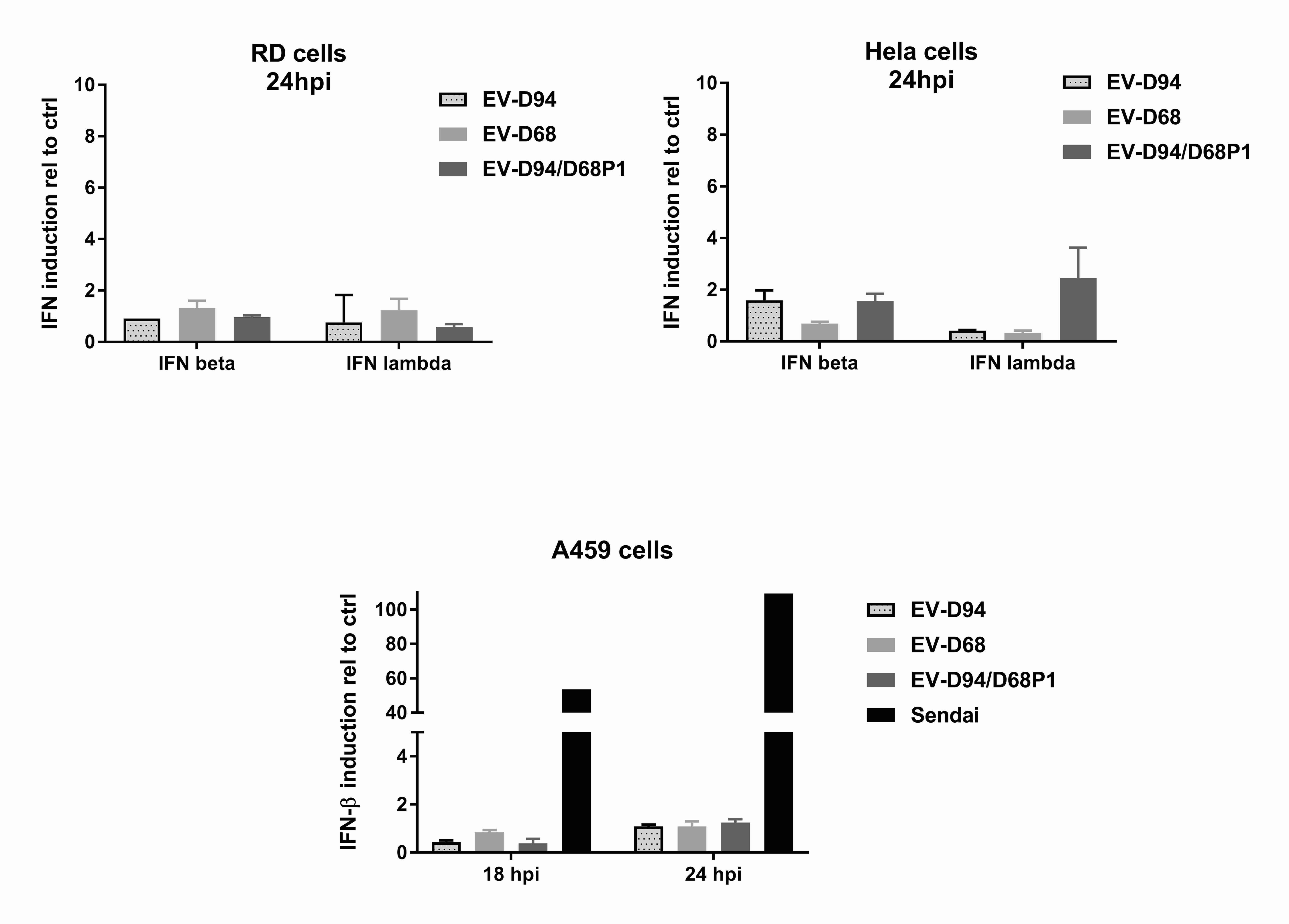

Supplement: S2 Fig — RD, Hela and A549 cells were infected with the indicated virus. For HeLa and RD cells, RNA was extracted from cell lysate 24 hpi and IFNβ and λ mRNAs were quantified by quantitative real-time PCR. The IFN Ct values were normalized to those of the RNAse P housekeeping gene (3416844, Applied Biosystems/ThermoFischer, USA) and relative quantification was calculated using the 2-ΔΔCt method. In A549/pr(IFN-β) GFP reporter cells [50], IFN induction was measured by FACS 18 and 24 hpi. Sendai virus was used as a positive control for IFNβ induction in A549 cells. hpi, hours post infection. (TIF) [file ppat.1006962.s002.tif]

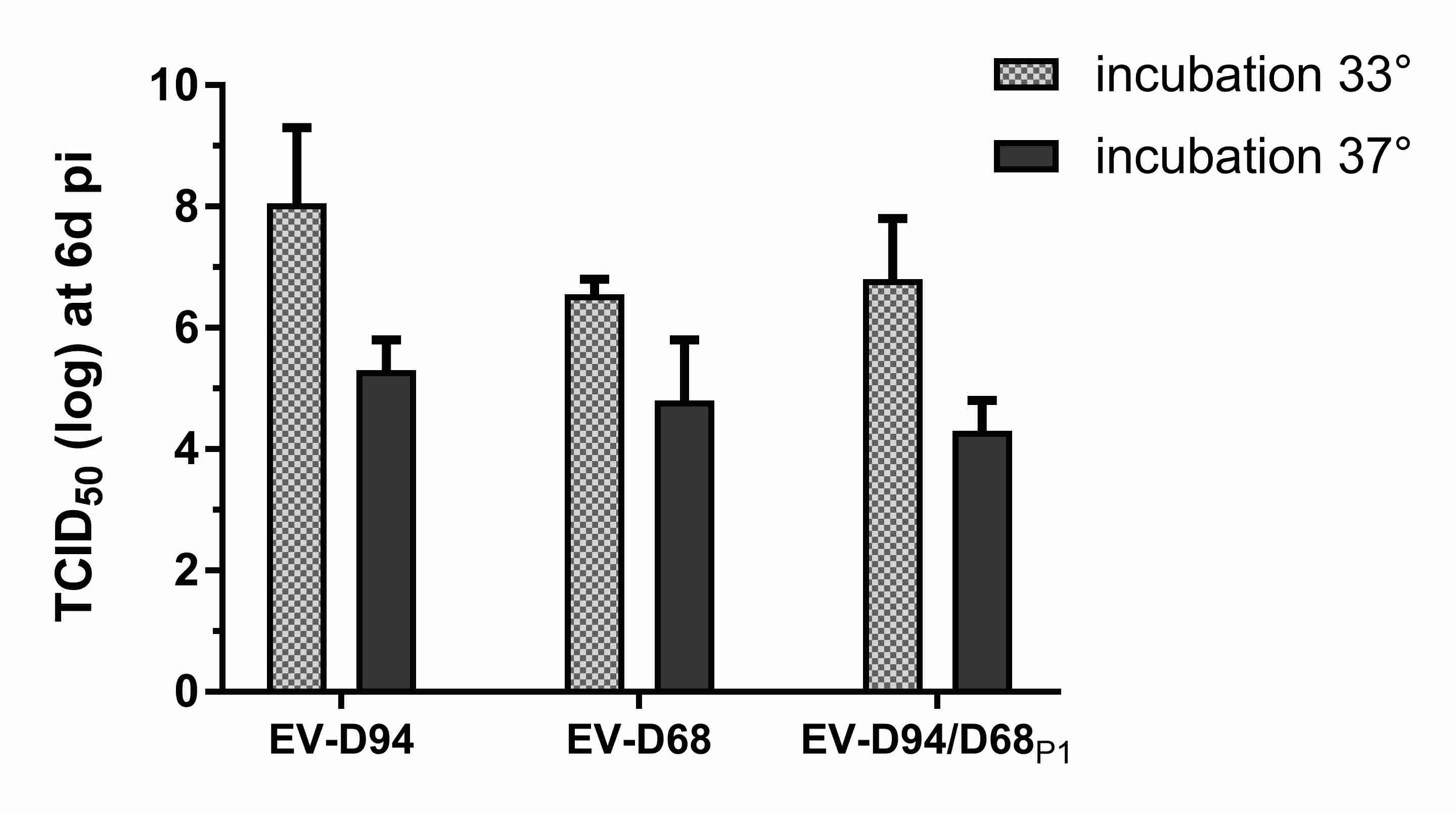

Supplement: S3 Fig — Viral stocks obtained after 3 passages in HeLa cells grown at 33°C were titrated at either 33°C or 37°C. Viral titers (expressed in log) were determined using the TCID50 method at 5 dpi. All viruses preferentially replicate at 33°C compared to 37°C. Experiments were run as biological duplicates. (TIF) [file ppat.1006962.s003.tif]

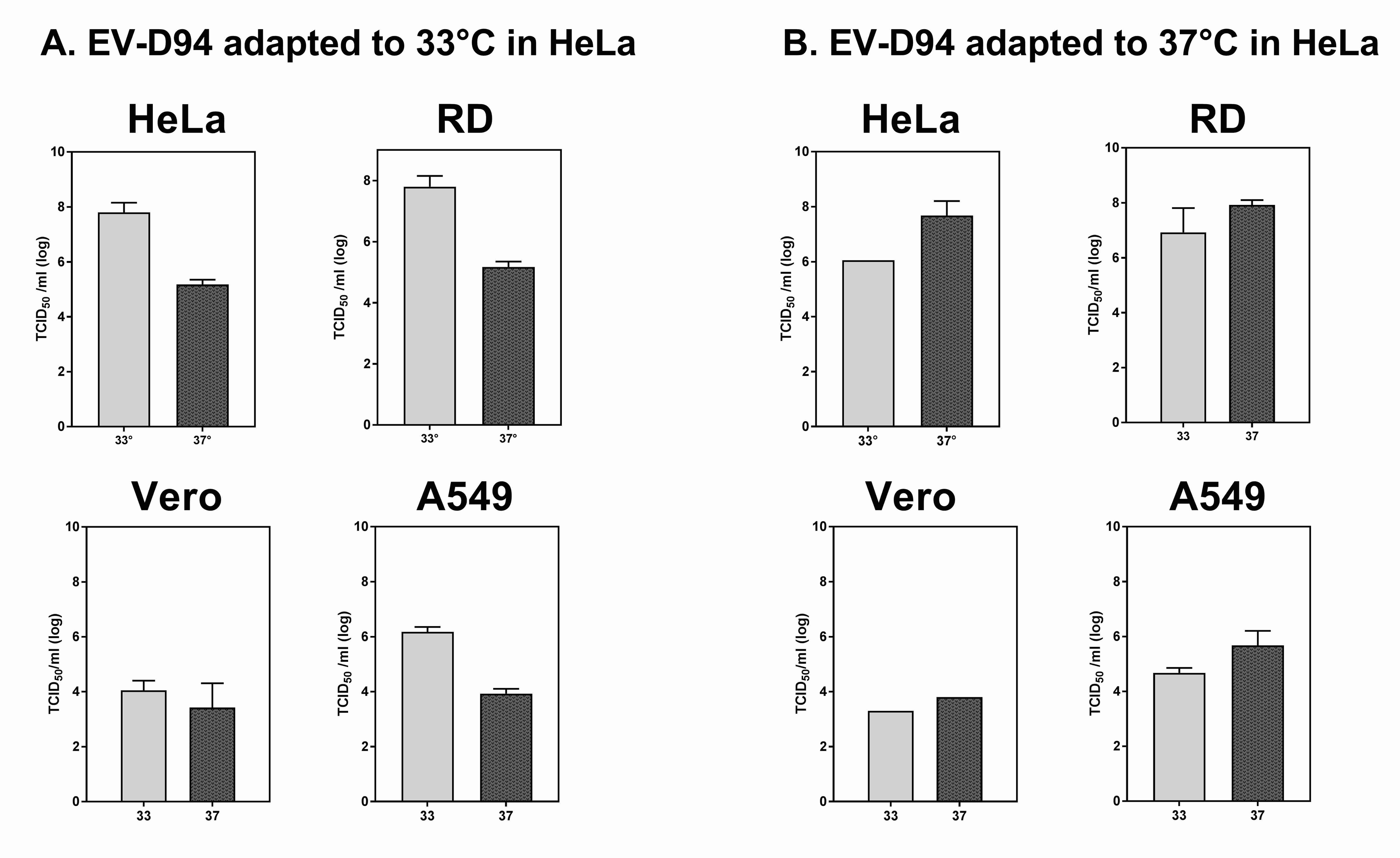

Supplement: S4 Fig — EV-D94 was transfected and passaged 6 times at 33°C or 37°C in HeLa to give rise to EV-D94 adapted to 33°C (A) and EV-D94 adapted to 37°C (B). Titration of the two stocks in HeLa, RD, Vero and A549 cells was performed at 33°C and 37°C to define their optimal growth temperature in each cell line. (TIF) [file ppat.1006962.s004.tif]

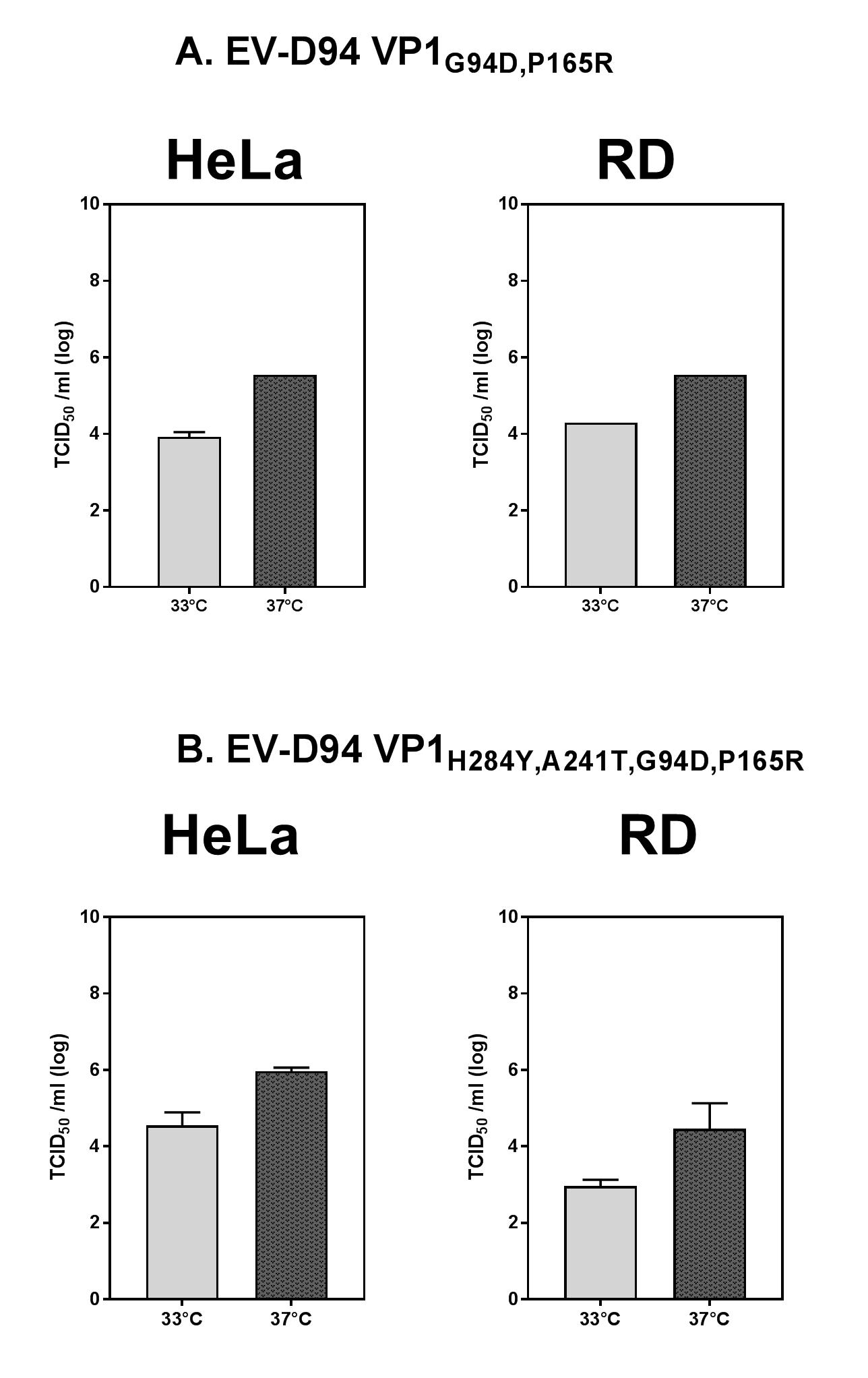

Supplement: S5 Fig — Replication of EV-D94 VP1G94D,P165R (A) and EV-D94 VP1H284Y,A241T,G94D, P165R (B) in HeLa and RD cells. (TIF) [file ppat.1006962.s005.tif]

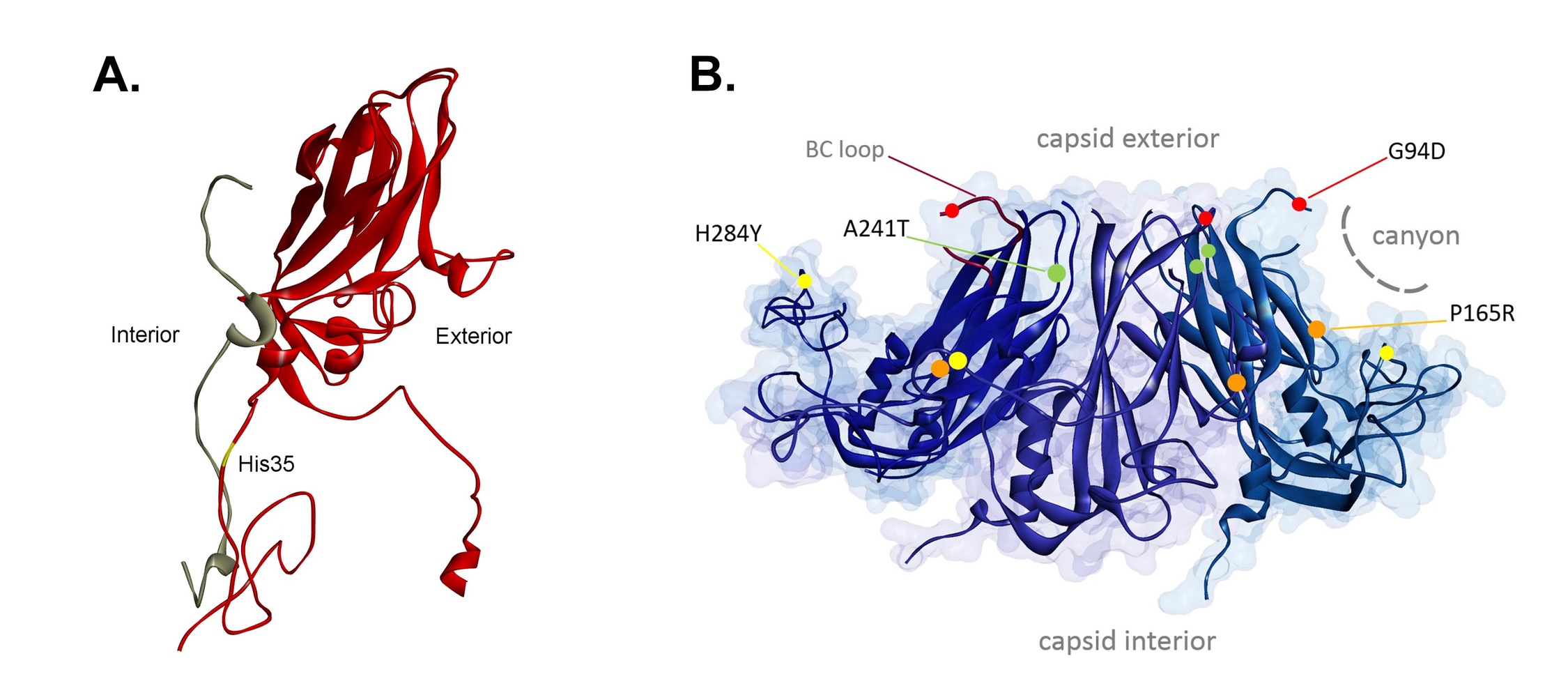

Supplement: S6 Fig — Mapping of the capsid residues mutated in EV-D94/D68P1 (A) or EV-D94 after amplification in cell culture at different temperatures (B). A. Residue VP3H35 (highlighted in yellow) is imaged in the context of VP3 (in red) and VP4 (in grey). The interior and exterior of the capsid are indicated. B. Mapping of residues mutated in EV-D94 based on homology with EV-D68. Amino acid G94D, P165R, A241T and H284Y in EV-D94 VP1 protein correspond to amino acid S82, L153, E227 and R270 in EV-D68. For A and B, mapping were performed on the available 3D structure of the EV-D68 capsid (PBD accession 4WM8 and 5BNP) thanks to Accelrys Discovery Studio Visualizer 3.5 (D.S. Visualizer, Accelrys Software Inc., San Diego, CA, USA 2012). (TIF) [file ppat.1006962.s006.tif]
