# Supplementary material for: Viral chimeras decrypt the role of enterovirus capsid proteins in viral tropism, acid sensitivity and optimal growth temperature
Source: PLoS Pathog. 2018 Apr 9;14(4):e1006962. doi: 10.1371/journal.ppat.1006962 (PMC5908207; doi:10.1371/journal.ppat.1006962)
Supplement: S1 Table — (DOCX) [file ppat.1006962.s007.docx]

**Supporting information:**

**Table S1: Primers used in this study.**

| Primer name | Sequence 5’-3’ |
| --- | --- |
| RV139 | TAATACGACTCACTATAGGTTAAAACAGCTCTGGGGTTGTTCCCACT |
| RV140 | TTTTTTTTTTTTTTTTTTTTTTTTTTTTTTTTTTTTTTTTTTTTTTTTTTGGTCCCCAAG |
| RV144 | GACCTGGGCGCCCATTGTTATAAATAAGTTTAAACTCTTTAAATG |
| RV146 | CCCAAAACCTGGACCTGTTGTAACGATGTCATTGGGCATGGTTGT |
| RV150 | TAATACGACTCACTATAGGTTAAAACAGCCTGTGGGTTGTTCCCACC |
| RV153 | ACCGAAACCAGGTCCGGTGGTTACTATGTTGTGAGGCATAGTTGT |
| RV155 | TTTTTTTTTTTTTTTTTTTTTTTTTTTTTTTTTTTTTTTTTTTTTTTTTTGATTCCCAAT |
| RV156 | GGGTAATACGACTCACTATAGGTTAAAACAGCTCTGGGGTTG |
| RV157 | GGGTAATACGACTCACTATAGGTT |
| RV158 | AACTTATTTATAACAATGGGCGCCCAGGTCTC |
| RV159 | CCCAAAACCTGGACCTGTTGTAACGATGTCATTGG |
| RV160 | GACATCGTTACAACAGGTCCAGGTTTTGGGGGAG |
| RV161 | TTTTTTTTTTTTTTTTTTTTTTTTGGTCCCCAAGTGA |
| RV164 | GGGTAATACGACTCACTATAGGTTAAAACAGCCTGTGGGTTG |
| RV167 | ACCGAAACCAGGTCCGGTGGTTACTATGTTGTGAGG |
| RV168 | AACATAGTAACCACCGGACCTGGTTTCGGTGGGG |
| RV169 | TTTTTTTTTTTTTTTTTTTTTTTTGATTCCCAATTAA |
| RV170 | ATGTAAGTGGTTTAATTGCCCGATATCAGGGCTGTCCCTCATAAG |
| RV171 | CCTGATATCGGGCAATTAAACCACTTACATGGAGCAG |
| RV192 | CAAGAACCATGTGACTGACACACAAAAAGTACAAC |
| RV193 | GTTGTACTTTTTGTGTGTCAGTCACATGGTTCTTG |
| RV194 | CCAGTAGGTGCTCGAACCCCAAAATCG |
| RV195 | CGATTTTGGGGTTCGAGCACCTACTGG |
| Ent1.81 | TTAAAACAGCTCTGGGGTTGTTC |
| Ent1.82 | ACCTACACAACCAGAAACTTCTACA |
| Ent1.83 | ACATTTTGTCCAAACATGCCTATG |
| Ent1.84 | AGTGGATCYCACTACAGGATG |
| Ent1.85 | TCAGAGGACTCACTGGGGAC |
| Ent1.86 | ACAACATAGTAACCACCGGTCC |
| Ent1.87 | ACAATGGGCGCCCAAGTTACTAG |
| Ent1.88 | GCATTACCTAGGTTTTGTATGTAATCAG |
| Ent1.89 | CAGCCTGTGGGTTGTTCC |
| Ent1.90 | AGTACAGGGTGGTGGTGGAA |
| Ent1.91 | TGCACTGTACATGGCATAGGA |
| Ent1.92 | GGTCTCACTTCCGCATGTTC |
| Ent1.93 | GCTGTCCCTCATAAGACGGA |
| Ent1.94 | TGCCCAATGACATCGTTACA |
| Ent2.21 | CCAGTCCACATAGATGGCTGAT |
| Ent2.22 | ACAACATAGTAACCACCGGTCC |
| Ent2.23 | TCCCCGCCTAATTTTTCTG |
| Ent2.24 | AGAGGCGTGCAATGCTCTTA |
| Ent2.25 | AGTACGCACCACTTTACGCA |
| Ent2.27 | TCATTTTCTGTGGCTAGGTGGT |
| Ent2.28 | AGTTCAAGTCCAAATCTCGCA |
| Ent2.29 | AGGTATCCCCACCGAGTTTC |
| Ent2.30 | CAGAGATGGTTTCAGTGAAGCC |
| Ent3.45 | CAACACATGCATCTGTTGGAG |
| Ent3.46 | GCGATCCAATTTGACTATAGTTATTTC |
| Ent3.47 | TGGAGAGATTTTTGCTTTTGATTA |
| Ent3.48 | AATCTATAAAAGATGTTTGGTGGGTG |
| Ent3.49 | TTTTGGTCCCCAAGTGACCA |
| Ent3.50 | ACGAAAGGTAGGTCAACTCCG |
| Ent3.52 | CATGTCTTTCAAAGCATAGGCA |
| Ent3.53 | GCTGGGAATATACGACAGGG |
| Ent3.54 | TCTCCAGTCTGGTTTGCTTG |
| Ent3.55 | ACAAATGAACCGAATGGCAC |
| Ent3.56 | GATTCCCAATTAACCAAAATTTACCTC |
| IFNfwd | GGACGCCTTGGAAGAGTCACT |
| IFNrev | AGAAGCCTCAGGTCCCAATTC |
| IFNprobe | AGTTGCAGCTCTCCTGTCTTCCCCG |
